# ER stress induces upregulation of transcription factor Tbx20 and downstream Bmp2 signaling to promote cardiomyocyte survival

**DOI:** 10.1016/j.jbc.2023.103031

**Published:** 2023-02-16

**Authors:** Shreya Das, Arunima Mondal, Chandrani Dey, Santanu Chakraborty, Rudranil Bhowmik, Sanmoy Karmakar, Arunima Sengupta

**Affiliations:** 1 Department of Life Science and Biotechnology, Jadavpur University, Kolkata, India; 2 Department of Life Sciences, Presidency University, Kolkata, India; 3 Bioequivalence Study Centre, Department of Pharmaceutical Technology, Jadavpur University, Kolkata, India

**Keywords:** ER stress, cardiomyopathy, Tbx20, proliferation, apoptosis

## Abstract

In the mammalian heart, fetal cardiomyocytes proliferate prior to birth; however, they exit the cell cycle shortly after birth. Recent studies show that adult cardiomyocytes re-enters the cell cycle postinjury to promote cardiac regeneration. The endoplasmic reticulum (ER) orchestrates the production and assembly of different types of proteins, and a disruption in this machinery leads to the generation of ER stress, which activates the unfolded protein response. There is a very fine balance between ER stress–mediated protective and proapoptotic responses. T-box transcription factor 20 (Tbx20) promotes embryonic and adult cardiomyocyte proliferation postinjury to restore cardiac homeostasis. However, the function and regulatory interactions of Tbx20 in ER stress–induced cardiomyopathy have not yet been reported. We show here that ER stress upregulates Tbx20, which activates downstream bone morphogenetic protein 2 (Bmp2)-pSmad1/5/8 signaling to induce cardiomyocyte proliferation and limit apoptosis. However, augmenting ER stress reverses this protective response. We also show that increased expression of *tbx20* during ER stress is mediated by the activating transcription factor 6 arm of the unfolded protein response. Cardiomyocyte-specific loss of Tbx20 results in decreased cardiomyocyte proliferation and increased apoptosis. Administration of recombinant Bmp2 protein during ER stress upregulates Tbx20 leading to augmented proliferation, indicating a feed-forward loop mechanism. In *in vivo* ER stress, as well as in diabetic cardiomyopathy, the activity of Tbx20 is increased with concomitant increased cardiomyocyte proliferation and decreased apoptosis. These data support a critical role of Tbx20-Bmp2 signaling in promoting cardiomyocyte survival during ER stress–induced cardiomyopathies.

In mammals, the developing heart is highly proliferative prior to birth, and it involves the interplay of multiple signaling pathways. However, after birth, the cardiomyocytes lose its plasticity, exit the cell cycle, its proliferative capacity dissipates, and the cells grow in size primarily by hypertrophy (1). In the neonates, post 1 week after birth, the cardiomyocytes become binucleated, express adult contractile protein isoforms, and lose its ability to regenerate (2, 3, 4). The notion that adult cardiomyocytes lose their capacity to proliferate because of cell cycle arrest was revoked by growing studies showing that resident adult myocardial cardiomyocyte re-enters cell cycle following myocardial injury by regulating key regulatory pathways (5).

T-box transcription factor 20 (Tbx20) is a member of the Tbx1 subfamily of T-box–containing genes and plays pivotal roles in development and maintenance of heart by driving cardiomyocyte proliferation (6). Loss of function of Tbx20 leads to unlooped and severely hypoplastic heart with embryonic lethality (7, 8, 9). Ablation of Tbx20 in adult cardiomyocytes leads to severe cardiomyopathy with arrhythmias and death (10). Gain of function of Tbx20 leads to increased cardiomyocyte proliferation in fetal heart development (11).

Endoplasmic reticulum (ER) is an organelle that mediates production and folding of different secretory and membrane proteins (12). Any sort of dysregulation in the machinery of the ER because of external factors or internal stimulus leads to accumulation of misfolded protein leading to generation of ER stress. ER stress activates the adaptive cellular response signaling cascade known as unfolded protein response (UPR), which consists of three pathways, activating transcription factor 6 (ATF6), inositol-requiring enzyme 1 alpha (IRE1α), and protein kinase RNA-activated-like ER kinase (PERK). The protective UPR is initially beneficial as it works for restoration of homeostasis; however, a severe ER stress leads to cell death *via* apoptosis. There is a very delicate balance between ER stress–induced prosurvival and proapoptosis (13). Tbx20 overexpression was previously shown to induce proliferation of cardiomyocytes during oxidative stress and hypoxia (14); however, its mechanistic role during ER stress–mediated cardiomyopathy is still elusive.

Our study for the first time identified the novel unknown function of Tbx20 that is able to directly enhance the protective responses of the UPR for restoration of ER homeostasis in the milieu of cardiac injury. Since ER stress have been implicated in the development of multiple cardiomyopathies; hence, we have used tunicamycin (Tun)-induced ER stress as our cardiomyopathy model system *in vivo* in order to look into a global phenomenon of ER stress–related cardiomyopathies. In the current study, we examine the function of Tbx20 and bone morphogenetic protein 2 (Bmp2) signaling during ER stress–induced cardiomyopathy. ER stress–mediated upregulation of Tbx20 resulted in increased cardiomyocyte proliferation *via* upregulating the Bmp2–pSmad1/5/8 signaling axis. Upregulation of Tbx20 also resulted in decreased cardiomyocyte apoptosis and fibrosis. However, increasing the intensity of ER stress or prolonging the ER stress resulted in decreased cardiomyocyte proliferation, increased apoptosis and fibrosis, thus disrupting cardiomyocyte homeostasis. In addition, we identify *tbx20* as a direct target of Atf6 during ER stress–mediated cardiomyopathy. Our study reported an elevated level of Bmp2 protein both during the initial stages as well as prolonged ER stress response in adult murine hearts, thus making it a potential biomarker candidate for early detection of ER stress–mediated cardiomyopathies.

## Results

### Tbx20 activity and Bmp2 activity are increased upon induction of ER stress in H9c2 cells *in vitro*

**Figure 1:**
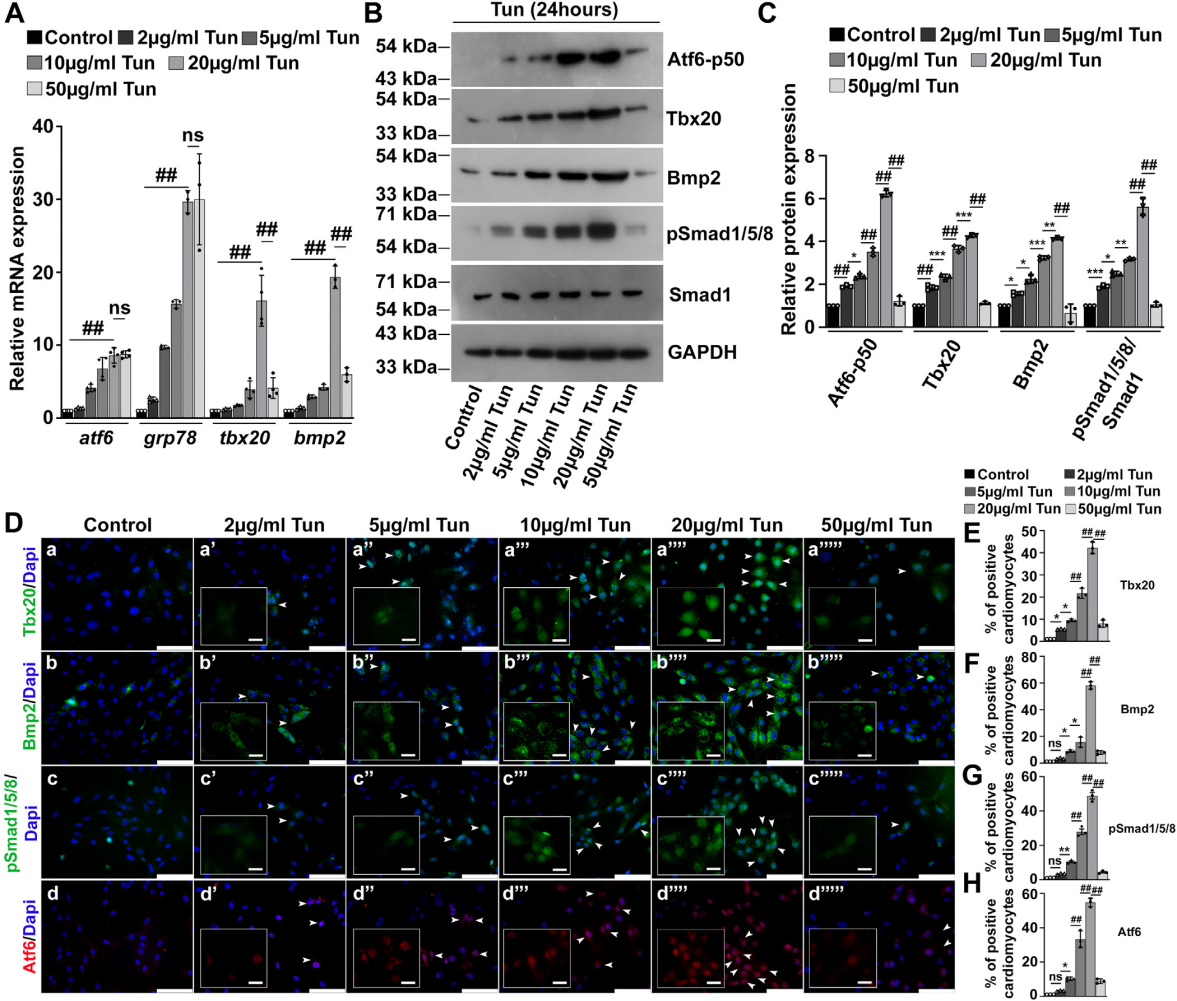
**T-box transcription factor 20 (Tbx20) and bone morphogenetic protein 2 (Bmp2) activity is increased upon endoplasmic reticulum (ER) stress induction *in vitro*.***A*, the expression of ER stress markers *atf6* and *grp78* is increased upon tunicamycin (Tun) treatment in H9c2 cells as determined by quantitative real-time PCR (qRT–PCR). The activity of Tbx20 and Bmp2 is concomitantly increased gradually upon ER stress induction (2 μg/ml Tun, 5 μg/ml Tun, 10 μg/ml Tun, and 20 μg/ml Tun). However, a 50 μg/ml Tun treatment resulted in decrease in the activity of Tbx20 and Bmp2 as determined by qRT–PCR. *B*, Western blot analysis showing a gradual increase in the expression of activating transcription factor 6 (Atf6)-p50 with increasing concentration of Tun. However, its expression decreased in 50 μg/ml Tun-treated cells. The expression of Tbx20, Bmp2, and its downstream cascade molecule pSmad1/5/8 also increased gradually up to a concentration of 20 μg/ml Tun. Increasing the concentration of Tun to 50 μg/ml resulted in significant decrease in the expression of Tbx20, Bmp2, and pSmad1/5/8. *C*, quantitative representation by ImageJ software of the proteins using three biological replicates from *B*. *D*, immunostaining analysis showing an increase in the expression of Tbx20, Bmp2, pSmad1/5/8, and Atf6 proteins upon increasing ER stress induction (a’, a’’, a’’’, and a’’’’), (b’, b’’, b’’’, and b’’’’), (c’, c’’, c’’’, and c’’’’), and (d’, d’’, d’’’, and d’’’’) as compared with control (a, b, c, and d), respectively. Increasing the intensity of ER stress by treatment with 50 μg/ml Tun however resulted in the decrease in expression of Tbx20 (a’’’’’), Bmp2 (b’’’’’), pSmad1/5/8 (c’’’’’), and Atf6 (d’’’’’). *Insets* in *B* show single-channel cropped images of indicated areas (*white arrows*). Scale bar of *main images* represent 50 μm. Scale bar of *inset* represents 20 μm. *E*–*H*, quantitative representation of *D*. Statistical significance was calculated by one-way ANOVA. Error bars represent SD from at least three biological replicates. ns, *p*: nonsignificant, ∗*p* < 0.05, ∗∗*p* < 0.005, ∗∗∗*p* < 0.0005, ^##^*p* < 0.0001; n ≥ 3 independent experiments.

The role of Tbx20 and Bmp2 signaling during ER stress–mediated cardiomyopathy has not been reported to date. To examine the expression profile of Tbx20 and Bmp2 upon induction of ER stress, we treated H9c2 cells with increasing concentration (2, 5, 10, 20, and 50 μg/ml) of ER stress–inducer Tun for 4, 8, 12, and 24 h. The 4 h time point did not show any significant change in the expression of Tbx20 and Bmp2 as compared with control (data not shown). Western blot analysis showed a gradual increase in the expression of Tbx20 and Bmp2 with increasing concentration of Tun with respect to control during 8 and 12 h (Fig. S1, *A* and *B*). Increased expression of cleaved form of Atf6 (Atf6-p50) at 24 h time point indicates the establishment of ER stress in our culture condition (Fig. 1, *B* and *C*). The expression of Atf6-p50 however decreased in the 50 μg/ml Tun-treated cells as compared with 20 μg/ml Tun-treated cells. Western blot analysis further showed a gradual increase in expression of Tbx20 up to a concentration of 20 μg/ml Tun during 24 h. However, increasing the concentration of Tun to 50 μg/ml resulted in significant decrease in the expression of Tbx20 (1.13 ± 0.07-fold) as compared with 20 μg/ml Tun-treated (4.26 ± 0.073-fold) cells. The Bmp2 signal transduction pathway was also increased gradually with increasing concentration of Tun. The expression of Bmp2 however decreased significantly in 50 μg/ml Tun-treated cells (0.66 ± 0.42-fold) as compared with 20 μg/ml Tun cell treatment (4.13 ± 0.05-fold; Fig. 1, *B* and *C*). Decrease in the expression of Bmp2 in the 50 μg/ml Tun-treated cells was accompanied by a decrease in its downstream signaling cascade molecule pSmad1/5/8 (1.06 ± 0.11-fold) as compared with 20 μg/ml Tun-treated cells (5.6 ± 0.39-fold; Fig. 1, *B* and *C*). Since the fold change increase in the expression of Tbx20 and Bmp2 was highest during 24 h as compared with 8 and 12 h, hence, this time point was used for further experiments.

Similar results were observed at mRNA level. The increased expression of ER stress markers *atf6* and *grp78* in the presence of 2, 5, 10, 20, and 50 μg/ml Tun, respectively, compared with control cell indicates ER stress induction (Fig. 1*A*). Interestingly, the expression of both *tbx20* and *bmp2* was also elevated gradually up to 20 μg/ml as compared with control. However, the expression of *tbx20* and *bmp2* was significantly decreased in 50 μg/ml Tun-treated cells as compared with 20 μg/ml Tun-treated cells (Fig. 1*A*).

To decipher whether other ER stress inducers have similar effect on the expression of Tbx20 and Bmp2, H9c2 cells were treated with DTT and thapsigargin (Tg). H9c2 cells were treated with DTT at a concentration of 1, 3, 5, and 10 mM for 24 h. The expression of Tbx20 was increased gradually up to 3 mM DTT-treated (2.5 ± 0.2-fold) cells as compared with control cells. However, it later decreased in 5 mM DTT- (0.82 ± 0.06-fold) and 10 mM DTT-treated (0.46 ± 0.04-fold) cells (Fig. S1, *D* and *E*). The expression of Bmp2 showed a similar pattern of increase up to 3 mM DTT treatment. The expression of Bmp2 in 5 mM DTT-treated cells is decreased; however, it was nonsignificant with respect to 3 mM. The expression of Bmp2 decreased significantly in 10 mM DTT-treated cells. H9c2 cardiomyocytes were treated with different concentrations (1.5, 3, 6, and 10 μM) of Tg. Tg treatment resulted in increased expression of both Tbx20 and Bmp2 up to 6 μM concentration. However, their expression later decreased in 10 μM Tg-treated cells (Fig. S1, *F* and *G*). Thus, all three ER stress inducers like Tun, DTT, and Tg result in increased expression of Tbx20 and Bmp2 up to a certain extent of ER stress. Increasing the ER stress further results in decrease in the expression of both Tbx20 and Bmp2.

Immunofluorescence technique was employed to determine the protein expression of the markers following ER stress induction. H9c2 cells treated with Tun showed significant increase in nuclear expression of Tbx20 up to 5.4 ± 0.18%, 9.5 ± 0.62%, 21.67 ± 2.3%, and 42.27 ± 2.6% when treated with 2 μg/ml Tun (Fig. 1, *D*a’ and *E*), 5 μg/ml Tun (Fig. 1, *D*a’’ and *E*), 10 μg/ml Tun (Fig. 1, *D*a’’’ and *E*), and 20 μg/ml Tun (Fig. 1, *B*a’’’’ and *E*), respectively, compared with control (1.06 ± 0.03%; Fig. 1, *D*a and *E*). However, its expression later decreased in 50 μg/ml Tun-treated cells (Fig. 1, *D*a’’’’’ and *E*). The immunoreactivity of Bmp2 also followed a similar trend of increase up to 20 μg/ml Tun (Fig. 1, *D*b’’’’ and *F*) as compared with control (Fig. 1, *D*b and *F*), which again decreased in 50 μg/ml Tun-treated (Fig. 1, *D*b’’’’’ and *F*) cells. Increase in Bmp2 signaling during ER stress was apparent by increased pSmad1/5/8-positive nuclei in 5 μg/ml Tun- (Fig. 1, *D*c’’ and *G*), 10 μg/ml Tun- (Fig. 1, *D*c’’’ and *G*), and 20 μg/ml Tun-treated (Fig. 1, *D*c’’’’ and *G*) cells as compared with control cells (Fig. 1, *D*c and *G*). Its expression later decreased in 50 μg/ml Tun-treated cells (Fig. 1, *D*c’’’’’ and *G*). The nuclear localization of Atf6 also increased up to 3.2 ± 0.3% in 2 μg/ml (Fig. 1, *D*d’ and *H*), 10.27 ± 1.0% in 5 μg/ml (Fig. 1, *D*d’’ and *H*), 33.27 ± 4.9% in 10 μg/ml (Fig. 1, *D*d’’’ and *H*), and up to 54.73 ± 2.5% in 20 μg/ml Tun-treated (Fig. 1, *D*d’’’’ and *H*) cells in comparison to control (Fig. 1, *D*d and *H*). However, its expression decreased to 8.86 ± 1.3% in 50 μg/ml Tun-treated (Fig. 1, *D*d’’’’’ and *H*) cells. These data further corroborate that Tbx20–Bmp2 signaling is elevated gradually with increasing ER stress, which is later decreased as the intensity of ER stress is increased in accordance with the cell death that is observed at higher concentration of Tun (Fig. S1*C*). The expression profile of Atf6 also correlated with the expression of Tbx20. These data thus suggest that the expression of Tbx20 and Bmp2 correlate with the viable status of the cells as well as the physiological status of ER stress.

### Atf6-mediated induction of Tbx20 promotes cardiomyocyte proliferation and limits cardiomyocyte apoptosis

**Figure 2:**
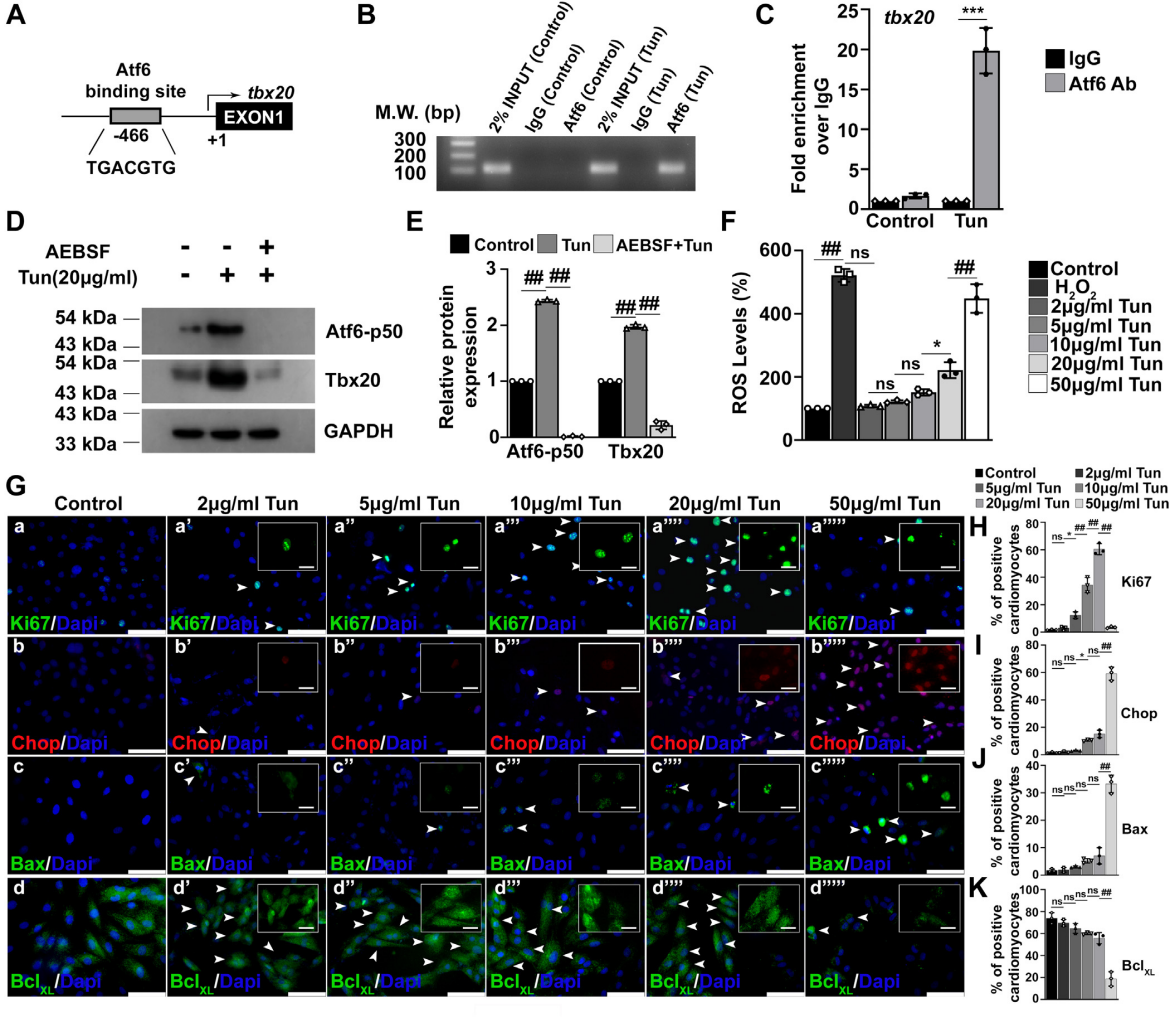
**During endoplasmic reticulum (ER) stress, activating transcription factor 6 (Atf6)-mediated upregulation of T-box transcription factor 20 (Tbx20) promotes cardiomyocyte proliferation and limits cardiomyocyte apoptosis in cultured H9c2 cardiomyocytes.***A*, the rat *tbx20* gene contains conserved canonical Atf6 DNA-binding sequence TGACGTG in the *tbx20* promoter region. *B*, chromatin immunoprecipitation (ChIP) analysis showed direct binding of Atf6 in *tbx20* promoter region. *C*, ChIP assay followed by quantitative RT–PCR (qRT–PCR) showed ∼19-fold enrichment of Atf6 binding to the *tbx20* promoter region during ER stress induction. *D*, Western blot analysis revealed pretreatment of H9c2 cardiomyocytes with 300 μM AEBSF followed by ER stress induction resulted in significant decrease in the expression of Atf6-p50. Inhibition of nuclear translocation of Atf6 was accompanied by concomitant decrease in the expression of Tbx20. *E*, quantitative representation by ImageJ software of the proteins using three biological replicates from *D*. *F*, level of ROS generation upon induction of ER stress showed a gradual increase in the level of ROS production by DCFDA method. Decrease in the expression of Tbx20 in 50 μg/ml tunicamycin (Tun)-treated cells was accompanied by significant rise in ROS levels as compared with 20 μg/ml Tun-treated cells. *G*, immunofluorescence staining revealed that increase in expression of Tbx20 during ER stress is accompanied with increased expression of proliferative marker Ki67 (a’’, a’’’, and a’’’’) as compared with control (a). However, a Tun concentration of 50 μg/ml resulted in decrease in cardiomyocyte proliferation marked by decreased expression of Ki67 (a’’’’’). Decrease in expression of Tbx20 is accompanied by increased cardiomyocyte apoptosis. The expression of apoptosis inducer Chop and proapoptotic marker Bax is increased upon 50 μg/ml Tun treatment (b’’’’’ and c’’’’’) in comparison to lower concentrations of Tun (b’, b’’, b’’’, and b’’’’) and (c’, c’’, c’’’, and c’’’’). The expression of antiapoptotic marker Bcl_XL_ is decreased during 50 μg/ml Tun treatment (d’’’’’) in comparison to lower concentrations of Tun (d’, d’’, d’’’, and d’’’’). *Insets* in *G* show single-channel cropped images of indicated areas (*white arrows*). Scale bar of *main images* represents 50 μm. Scale bar of *inset* represents 20 μm. *H*–*K*, quantitative representation of *G*. Statistical significance was calculated by one-way ANOVA. Error bars represent SD from n = 3 independent biological replicates. ns, *p*: nonsignificant, ∗*p* < 0.05, ∗∗*p* < 0.005, ∗∗∗*p* < 0.0005, ^##^*p* < 0.0001. AEBSF, 4-(2-aminoethyl) benzenesulfonyl fluoride hydrochloride; DCFDA, 2′,7′-dichlorofluorescin diacetate; ROS, reactive oxygen species.

Our study has shown an increase in the expression of Tbx20 during ER stress induced by Tun, DTT, and Tg; however, the mechanism behind this increase in the expression is still elusive. Since the increase in the expression of Tbx20 followed a similar pattern of increase to that of Atf6-p50 and with decrease in the expression of Atf6-p50 in 50 μg/ml Tun-treated cells, the expression of Tbx20 also decreased; this led us to speculate a possible role of Atf6 in the upregulation of Tbx20 during ER stress. Atf6, which is a basic leucine zipper family of transcription factor, was previously shown to bind to canonical UPRE TGACGTGG/A of various genes in order to transcribe them (15). Atf6 was shown to impart its cardioprotective effect in the direction of prosurvival by ameliorating the extent of ER stress, and it also has role in compensatory myocyte growth. It was also shown to confer global protection of cardiomyocytes from ischemia/reperfusion injury by reprogramming cellular proteostasis (16). Atf6 was also shown to play vital role in maintaining homeostasis of cardiomyocytes under both pathological and physiological states. In one study, chromatin immunoprecipitation (ChIP)-Seq analysis was performed to identify the putative targets of Atf6 (17). This study identified Tbx20 as one of the possible targets of Atf6. Hence, *tbx20* genomic sequences were examined for Atf6 binding consensus sequence. Bioinformatics analysis revealed the presence of canonical UPRE TGACGTG binding sequence for Atf6 in the promoter of rat *tbx20* gene (Fig. 2*A*). To examine whether Atf6 controls the expression of *tbx20* gene through direct binding, H9c2 cells were treated with 20 μg/ml Tun, and the DNA-binding ability of Atf6 to *tbx20* promoter was performed using ChIP assay. The results revealed direct binding of Atf6 to the promoter region of *tbx20* in ER stress–induced H9c2 cells (Fig. 2*B*). Real-time analysis revealed that in Tun-treated cardiomyocytes, Atf6 binds to the promoter of *tbx20* with 19.85 ± 2.8-fold enrichment over immunoglobulin G (IgG) controls (Fig. 2*C*).

In order to corroborate the direct binding of Atf6 to *tbx20* promoter, the H9c2 cardiomyocytes were treated with Atf6-specific inhibitor 4-(2-aminoethyl) benzenesulfonyl fluoride hydrochloride (AEBSF), which blocks the cleavage of Golgi-membrane-bound Atf6, thereby blocking its nuclear translocation followed by treatment with Tun (20 μg/ml). Western blot analysis showed decreased nuclear localization of Atf6-p50 by 0.01 ± 0.01-fold upon treatment with AEBSF followed by Tun as compared with Tun treatment alone (2.43 ± 0.02-fold; Fig. 2, *D* and *E*). Decrease in the nuclear translocation of Atf6 because of AEBSF treatment was accompanied by subsequent decrease in the expression of Tbx20 (0.21 ± 0.07-fold) during ER stress as compared with ER stress induction alone (1.97 ± 0.03-fold; Fig. 2, *D* and *E*). Therefore, our study showed that increase in the expression of Tbx20 during ER stress was mediated by Atf6 because of its direct DNA-binding ability to the promoter region of *tbx20* gene.

Tun acts by inhibiting N-linked glycosylation, thus resulting in improper maturation of proteins (18). Increasing ER stress results in increased disruption of the disulphide bonds resulting in increased reactive oxygen species (ROS) generation (19, 20). We therefore looked into the ROS levels in our study. Increasing the ER stress resulted in a gradual increase in ROS generation. However, at a Tun concentration of 50 μg/ml when the expression of Tbx20 was reduced, the level of ROS increased drastically (Fig. 2*F*). Previous studies have shown that overexpression of Tbx20 results in decreased levels of ROS (21). Thus, our study showed that ER stress–induced increase in the Tbx20 restricts ROS generation; however, decrease in the expression of Tbx20 results in drastic increase in ROS generation during ER stress.

Tbx20 was shown to promote fetal cardiomyocyte proliferation and inhibit cardiomyocyte apoptosis (22, 23). To decipher the role of increased Tbx20 during ER stress–induced cardiomyopathy, we looked into the expression profile of proliferative (Ki67) and apoptotic markers (Chop and Bax) post ER stress induction in H9c2 cells. The Ki67-positive nuclei increased gradually up to 12.4 ± 2.4% in 5 μg/ml Tun (Fig. 2, *G*a’’ and *H*), 34.3 ± 5.5% in 10 μg/ml Tun (Fig. 2, *G*a’’’ and *H*), and 60.43 ± 4.04% in 20 μg/ml Tun-treated (Fig. 2, *G*a’’’’ and *H*) cells, respectively, as compared with control (Fig. 2, *G*a and *H*). However, it later decreased to 3.5 ± 0.6% in 50 μg/ml Tun-treated cells (Fig. 2, *G*a’’’’’ and *H*). The increased expression for Ki67 correlated with that of Tbx20, with highest cardiomyocyte proliferation in 20 μg/ml Tun-treated cells where the expression of Tbx20 and Bmp2 was also the highest. Tg treatment also resulted in increased cardiomyocyte proliferation as marked by increased Ki67-positive cells at a concentration of 3 μM Tg-treated cells (Fig. S2, *A*a’ and *B*) where the expression of Tbx20 and Bmp2 was also increased (Fig. S1, *F* and *G*) as compared with control (Fig. S2, *A*a and *B*). However, it later decreased at a concentration of 10 μM Tg (Fig. S2, *A*a’’ and *B*). Thus, Tbx20 works during ER stress by increasing cardiomyocyte proliferation.

Conversely, increased expression of Tbx20 limits cardiomyocyte apoptosis. Chop plays a pivotal role in ER stress–mediated apoptosis by downregulating the expression of antiapoptotic marker Bcl-_XL_ and augmenting the expression of proapoptotic marker Bax (24). The expression of Chop increased significantly up to 59.13 ± 4.8% in 50 μg/ml Tun-treated (Fig. 2, *G*b’’’’’ and *I*) cells as compared with 20 μg/ml Tun (15.17 ± 2.8%; Fig. 2, *G*b’’’’ and *I*). Tg treatment also resulted in significant increase in the expression of Chop at a concentration of 10 μM Tg (Fig. S2, *A*b’’ and *B*) where the expression of Tbx20 was decreased (Fig. S1, *F* and *G*) as compared with control (Fig. S2, *A*b and *B*) and 3 μM Tg-treated (Fig. S2, *A*b’ and *B*) cells. Increased expression of Chop resulted in increased immunoreactivity of Bax up to 33.23 ± 3.2% in 50 μg/ml Tun-treated (Fig. 2, *G*c’’’’’ and *J*) cells as compared with 20 μg/ml Tun (7.03 ± 2.9%; Fig. 2, *G*c’’’’ and *J*), 10 μg/ml Tun (5.2 ± 0.8%; Fig. 2, *G*c’’’ and *J*), and 5 μg/ml Tun (2.9 ± 0.37%; Fig. 2, *G*c’’ and *J*), 2 μg/ml Tun (1.9 ± 0.85%; Fig. 2, *G*c’ and *J*), and control (1.7 ± 0.62%; Fig. 2, *G*c and *J*) cells. On the contrary, the expression of antiapoptotic marker Bcl-_XL_ decreased to 18.67 ± 6.5% in 50 μg/ml Tun-treated (Fig. 2, *G*d’’’’’ and *K*) cells in comparison to 20 μg/ml Tun (55.83 ± 5.1%; Fig. 2, *G*d’’’’ and *K*), 10 μg/ml Tun (60 ± 1.0%; Fig. 2, *G*d’’’ and *K*), 5 μg/ml Tun (64.6 ± 4.5%; Fig. 2, *G*d’’ and *K*), 2 μg/ml Tun (69.67 ± 3.1%; Fig. 2, *G*d’ and *K*), and control (74 ± 5.0%; Fig. 2, *G*d and *K*) cells. Thus, ER stress–induced expression of Tbx20 results in activation of Bmp2–pSmad1/5/8 signaling that increases cardiomyocyte proliferation. However, after a certain threshold, ER stress eventually leads to decrease in the expression of Tbx20 with concomitant increase in cardiomyocyte apoptosis and Tbx20 fails to impart its protective role.

### Tbx20 is necessary, and it acts upstream of Bmp2–pSmad1/5/8 signaling in protecting cardiomyocytes against Tun-induced ER stress

**Figure 3:**
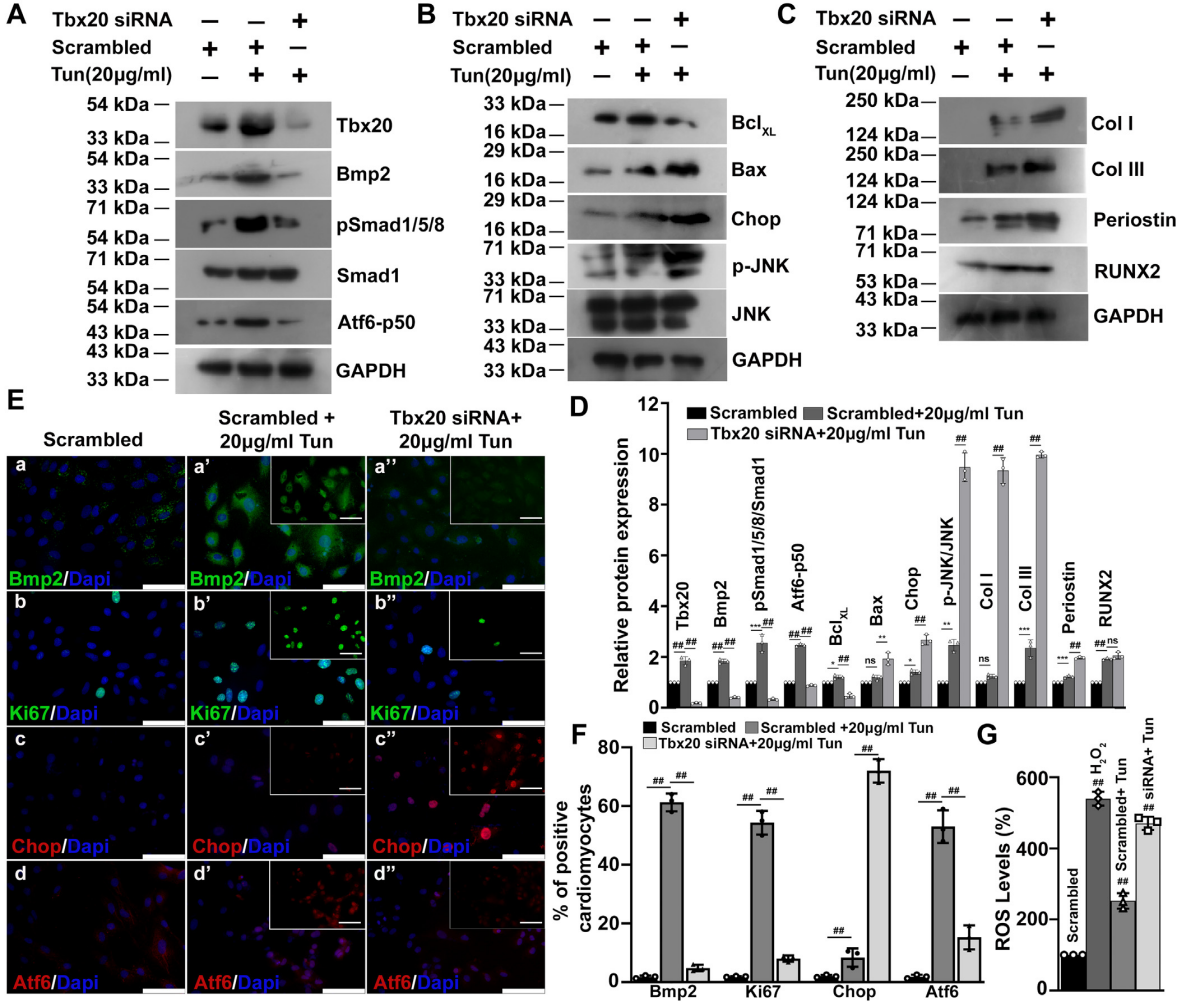
**T-box transcription factor 20 (Tbx20) acts upstream of Bmp2–pSmad1/5/8 signaling in protecting cultured H9c2 cardiomyocytes against endoplasmic reticulum (ER) stress.***A*, Western blot analysis showed a decrease in the expression of Tbx20 upon knockdown with Tbx20 siRNA followed by ER stress induction as compared with ER stress induction alone. Knockdown of Tbx20 followed by ER stress induction resulted in significant decrease in the expression of Bmp2, its downstream signal transducer pSmad1/5/8 and Atf6. *B*, knockdown of Tbx20 followed by ER stress induction resulted in increased cardiomyocyte apoptosis as marked by increased expression of Bax, Chop, p-JNK, and decreased expression of Bcl_XL_ as compared with tunicamycin (Tun) treatment alone. *C*, knockdown of Tbx20 followed by ER stress induction also resulted in increased expression of fibrotic genes Collagen I (Col I), Collagen III (Col III), and Periostin. The expression of calcification marker (RUNX2) increased significantly from control group; however, its expression between knockdown group and ER stress induction alone group remained unchanged. *D*, quantitative representation by ImageJ software of the proteins using three biological replicates from *A*–*C*. *E*, immunofluorescence staining showed siRNA-mediated knockdown of Tbx20 followed by ER stress induction (20 μg/ml Tun) resulted in decreased expression of Bmp2 (a’’) as compared with 20 μg/ml Tun treatment alone (a’) and control cells (a). Knockdown of Tbx20 followed by Tun treatment is accompanied by decreased cardiomyocyte proliferation marked by decreased expression of Ki67 (b’’) and increased apoptosis marked by increased expression of Chop (c’’) as compared with Tun treatment alone (b’ and c’) and control cells (b and c), respectively. Knockdown of Tbx20 followed by Tun treatment resulted in decreased expression of Atf6 (d’’) as compared with Tun treatment alone (d’) and control cells (d). *Insets* in *E* show single-channel images of respective makers. Scale bar of *main images* and *insets* represents 50 μm. *F*, quantitative representation of *E*. *G*, measurement of reactive oxygen species (ROS) levels showed a significant increase upon knockdown of Tbx20 followed by ER stress induction. Statistical significance was calculated by one-way ANOVA. Error bars represent SD from three independent biological replicates (n = 3). ns, *p*: nonsignificant, ∗*p* < 0.05, ∗∗*p* < 0.005, ∗∗∗*p* < 0.0005, ^##^*p* < 0.0001. Atf6, activating transcription factor 6; Bmp2, bone morphogenetic protein 2; p-JNK, phosphorylated form of c-Jun N-terminal kinase; Tbx20, T-box transcription factor 20.

Our study has shown that Tbx20 promotes cardiomyocyte proliferation during ER stress by activating Bmp2–pSmad1/5/8 signaling axis. Previous studies have shown that Tbx20 acts upstream of Bmp2 during heart development (22, 25, 26). To determine the molecular hierarchy between Tbx20–Bmp2–Smad1/5/8 signaling axis and to decipher the mode of action by which Tbx20 protects the cardiomyocyte against ER stress, Tun-treated H9c2 cells were pretreated with Tbx20-specific siRNA. Treatment of H9c2 cells with 100 nM Tbx20 siRNA resulted in 73% reduction in the expression of endogenous level of Tbx20 (Fig. S2, *H* and *I*). Western blot analysis showed that pretreatment of H9c2 cells with Tbx20-specific siRNA followed by ER stress induction resulted in significant decrease in the expression of Tbx20 (0.18 ± 0.02-fold) as compared with Tun treatment alone (1.84 ± 0.15-fold) (Fig. 3, *A* and *D*). Knockdown of Tbx20 followed by ER stress induction resulted in concomitant decrease in the expression of Bmp2 (0.4 ± 0.03-fold) and its downstream signaling molecule pSmad1/5/8 (0.33 ± 0.03-fold) as compared with Tun treatment alone (Fig. 3, *A* and *D*). These data suggested that Tbx20 functions upstream of Bmp2 in imparting protection during ER stress.

Next, we looked in the mode of action of Tbx20 in imparting protection against ER stress–induced cardiomyopathy. Knockdown of Tbx20 followed by ER stress induction resulted in significant increase in the expression of proapoptotic marker Bax (2 ± 0.24-fold) as compared with Tun treatment alone (1.18 ± 0.09-fold; Fig. 3, *B* and *D*). However, the expression of antiapoptotic marker Bcl_XL_ decreased significantly (0.4 ± 0.09-fold) upon knockdown of Tbx20 (Fig. 3, *B* and *D*). Knockdown of Tbx20 also resulted in significant increase in the expression of apoptosis inducer Chop (2.67 ± 0.19-fold) as compared with Tun treatment alone (1.4 ± 0.08-fold; Fig. 3, *B* and *D*). Phosphorylated form of c-Jun N-terminal kinase (p-JNK) was shown to induce the expression of proapoptotic genes during ER stress. JNK was previously shown to abrogate the antiapoptotic effect of Bcl2 during ER stress (27). Tbx20 knockdown also resulted in significant increase in the expression of p-JNK (9.4 ± 0.56-fold) as compared with Tun treatment alone (2.4 ± 0.22-fold). Thus, our study showed that Tbx20 imparts its protection against ER stress by decreasing cardiomyocyte apoptosis.

Since previous studies have shown the involvement of ER stress during fibrosis and calcification (28, 29), therefore, we looked into the expression of fibrotic markers Collagen I, Collagen III, Periostin, and calcification marker RUNX2. The expression of all the three fibrotic markers increased significantly upon knockdown of Tbx20 followed by Tun treatment as compared with Tun treatment alone (Fig. 3, *C* and *D*). The expression of calcification marker RUNX2 however remained unchanged upon knockdown of Tbx20 (Fig. 3, *C* and *D*). Immunostaining study showed that knockdown of Tbx20 followed by 20 μg/ml Tun treatment resulted in decreased expression of Bmp2 (4.8 ± 1.0%; Fig. 3, *E*a’’ and *F*) as compared with 20 μg/ml Tun treatment alone (61.27 ± 3.0%; Fig. 3, *E*a’ and *F*). Next, we checked the proliferation profile of the cardiomyocytes following knockdown of Tbx20 and subsequent ER stress induction. siRNA-mediated knockdown of Tbx20 followed by 20 μg/ml Tun treatment resulted in decreased immunoreactivity of Ki67 (8.03 ± 1.0%; Fig. 3, *E*b’’ and *F*) as compared with 20 μg/ml Tun treatment alone (54.33 ± 4.0%; Fig. 3, *E*b’ and *F*).

Knockdown of Tbx20 followed by 20 μg/ml Tun administration caused increased cardiomyocyte apoptosis. The expression of Chop increased up to 72 ± 4.0% (Fig. 3, *E*c’’ and *F*) upon knockdown of Tbx20 followed by Tun (20 μg/ml) treatment as compared with 20 μg/ml Tun treatment alone (8.14 ± 3.1%; Fig. 3, *E*c’ and *F*). Thus, decrease in the expression of Tbx20 followed by ER stress induction is accompanied by increased cardiomyocyte apoptosis. Similarly, knockdown of Tbx20 followed by 20 μg/ml Tun treatment also resulted in decreased expression of Atf6 (Fig. 3, *E*d’’ and *F*) as compared with 20 μg/ml Tun (Fig. 3, *E*d’ and *F*) treatment alone. Knockdown of Tbx20 followed by ER stress induction resulted in significant decrease in the expression of both total as well as cleaved form of Atf6 as evidenced by Western blot and immunostaining analysis. This led us to speculate a possible role of Tbx20 in maintaining the pool of Atf6 in cells during ER stress. ChIP assay followed by PCR analysis revealed that Tbx20 binds to *atf6* promoter and induces its activity (Fig. S2, *E* and *F*). In 20 μg/ml Tun-treated cardiomyocytes, Tbx20 binds to the promoter of *atf6* with 7.3 ± 1.2-fold enrichment over IgG controls (Fig. S2*G*). Hence, these data suggest that Tbx20 directly binds to and induces the expression of *atf6* during ER stress–mediated cardiomyopathy, thus maintaining the pool of *atf6* during stressed conditions.

Knockdown of Tbx20 followed by ER stress induction also resulted in significant increase in the level of ROS as compared with Tun treatment alone (Fig. 3*G*). Thus, this result strengthened the previous observation for the involvement of Tbx20 in restricting ROS generation during ER stress.

Together, these data suggest that Tbx20 is located upstream of Bmp2–pSmad1/5/8 signaling axis, and it is necessary in imparting protection against ER stress by increasing cardiomyocyte proliferation and decreasing cardiomyocyte apoptosis and fibrosis, which together results in restoration of cardiomyocyte homeostasis.

### Tbx20–Bmp2 signaling acts in a feed-forward loop mechanism in protecting cells against Tun-induced ER stress

**Figure 4:**
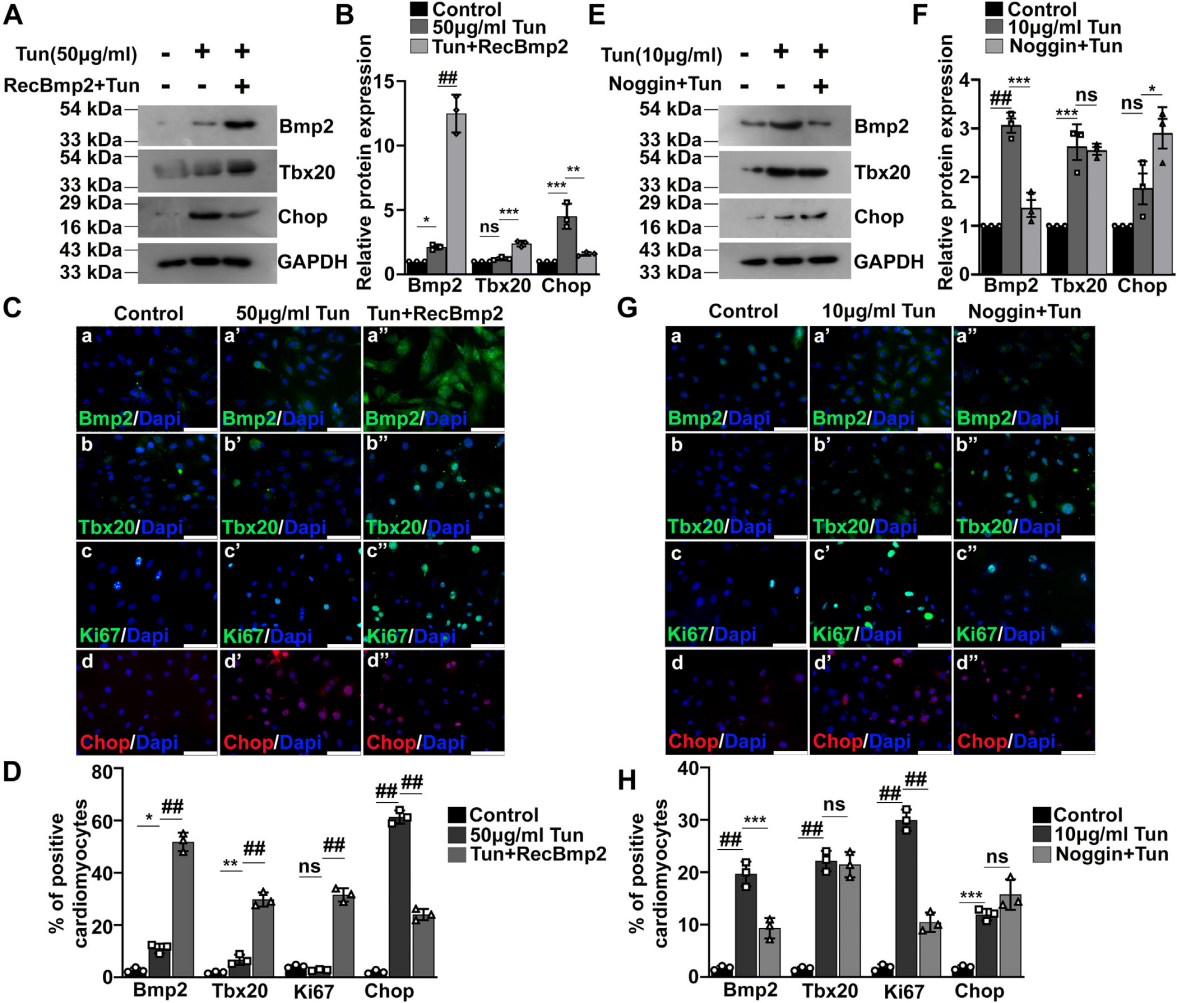
**Tbx20–Bmp2 signaling acts in a feed-forward loop mechanism to protect cultured H9c2 cardiomyocytes against endoplasmic reticulum (ER) stress.***A*, Western blot analysis of H9c2 cells treated with 50 μg/ml tunicamycin (Tun) followed by administration of recombinant Bmp2 (RecBmp2) showed significant increase in the expression of Bmp2 even during increased ER stress. Administration of RecBmp2 protein following ER stress induction resulted in significant increase in the expression of Tbx20 as compared with 50 μg/ml Tun-treated group. Treatment of the ER-stressed cells with RecBmp2 also resulted in decrease in the expression of apoptotic marker Chop. *B*, quantitative representation by ImageJ software of the proteins using three biological replicates from *A*. *C*, immunofluorescence staining showed that RecBmp2 treatment following ER stress induction (50 μg/ml Tun) resulted in increase in the expression of Bmp2 (a’’) and Tbx20 (b’’) in comparison to 50 μg/ml Tun treatment alone (a’ and b’) and control cells (a and b), respectively. Increase in Bmp2 expression is accompanied by increased expression of Ki67 (c’’) and decreased expression of Chop (d’’) as compared with 50 μg/ml Tun treatment alone (c’ and d’) and control cells (c and d), respectively. *D*, quantitative representation of panels in *C*. *E*, Western blot analysis of H9c2 cells treated with Bmp2 inhibitor Noggin followed by ER stress induction (10 μg/ml Tun) caused significant decrease in the expression of Bmp2 as compared with ER stress induction group alone. Treatment with Noggin followed by ER stress induction however caused no significant change in the expression of Tbx20 from ER stress induction-alone group. *F*, quantitative representation by ImageJ software of the proteins using three biological replicates from *E*. *G*, immunostaining of H9c2 cells treated with Bmp2 inhibitor Noggin followed by Tun treatment (10 μg/ml Tun) resulted in decrease in the expression of Bmp2 (a’’) with reference to 10 μg/ml Tun treatment alone (a’) and control cells (a). Noggin treatment followed by ER stress induction caused no significant change in the expression of Tbx20 (b’’) as compared with ER stress induction alone (b’). Noggin administration followed by Tun treatment resulted in significant decrease in the expression of proliferative marker Ki67 (c’’) as compared with Tun treatment alone group (c’). The expression of apoptosis inducer Chop however remained unchanged between Noggin administered group (d’’) as compared with Tun treatment alone (d’). *H*, quantitative representation of panels in *G*. Scale bar represents 50 μm. Statistical significance was calculated by one-way ANOVA. Error bars represent SD from three independent biological replicates (n = 3); ns, *p*: nonsignificant, ∗*p* < 0.05, ∗∗*p* < 0.005, ∗∗∗*p* < 0.0005, ^##^*p* < 0.0001. Bmp2, bone morphogenetic protein 2; Tbx20, T-box transcription factor 20.

To decipher the regulatory relationship between Tbx20 and Bmp2, H9c2 cardiomyocytes were treated with 50 μg/ml Tun followed by treatment with recombinant Bmp2 (RecBmp2) protein. Treatment of H9c2 cells with 200 ng/ml RecBmp2 protein resulted in 74.2% increase in the expression of endogenous level of Bmp2 (Fig. S2, *J* and *K*). Western blot analysis showed a significant increase in the expression of Bmp2 (12.49 ± 1.4-fold) upon treatment of RecBmp2 protein following ER stress induction as compared with ER stress induction alone (2.1 ± 0.21-fold; Fig. 4, *A* and *B*). RecBmp2 treatment following ER stress induction also resulted in increased expression of Tbx20 (2.3 ± 0.23-fold) as compared with 50 μg/ml Tun treatment alone (1.2 ± 0.12-fold; Fig. 4, *A* and *B*). The expression of apoptotic marker Chop also decreased significantly upon administration of RecBmp2 protein as compared with ER stress induction alone (Fig. 4, *A* and *B*).

Immunofluorescence study showed that the expression of Bmp2 was increased significantly (51.83 ± 3.5%; Fig. 4, *C*a’’ and *D*) in RecBmp2 and Tun group as compared with Tun treatment alone (11.07 ± 1.8%; Fig. 4, *C*a’ and *D*). The expression of Tbx20 was significantly augmented by approximately 29.80 ± 2.6% (Fig. 4, *C*b’’ and *D*) upon RecBmp2 and Tun treatment relative to Tun treatment alone (12.33 ± 1.6%; Fig. 4, *C*b’ and *D*). Ki67 immunoreactivity, which is indicative of cardiac proliferation, also increased by approximately 31.57 ± 2.5% (Fig. 4, *C*c’’ and *D*) upon RecBmp2 and Tun treatment as compared with Tun treatment alone (2.8 ± 0.3%; Fig. 4, *C*c’ and *D*). However, RecBmp2 and Tun treatment resulted in significant decrease in the expression of apoptosis inducer Chop by approximately 24.03 ± 2.1% (Fig. 4, *C*d’’ and *D*) as compared with only Tun treatment (61.37 ± 2.5%; Fig. 4, *C*d’ and *D*).

To further corroborate our results, the H9c2 cells were pretreated with Noggin followed by induction of ER stress. Treatment of H9c2 cells with 200 ng/ml Noggin protein resulted in 67% reduction in the expression of endogenous level of Bmp2 (Fig. S2, *L* and *M*). Pretreatment of 10 μg/ml Tun-treated H9c2 cardiomyocytes with Bmp2 inhibitor Noggin resulted in significant decrease in the expression of Bmp2 (1.36 ± 0.3-fold) as compared with ER stress induction alone (3.06 ± 0.25-fold; Fig. 4, *E* and *F*). However, Noggin treatment resulted in no significant change in the expression of Tbx20 (2.5 ± 0.13-fold) as compared with ER stress induction alone (2.62 ± 0.45-fold; Fig. 4, *E* and *F*).

Immunostaining analysis also corroborated with the Western blot results. Pretreatment of 10 μg/ml Tun-treated H9c2 cardiomyocytes with Bmp receptor inhibitor Noggin caused no significant change in the expression of Tbx20 (Fig. 4, *G*b’’ and *H*) as compared with 10 μg/ml Tun treatment alone (Fig. 4, *G*b’ and *H*). However, inhibition of Bmp2 resulted in significant attenuation in cardiomyocyte proliferation marked by reduced Ki67-positive nuclei by approximately 10.47 ± 1.8% (Fig. 4, *G*c’’ and *H*) relative to Tun treatment alone (29.97 ± 2.0%; Fig. 4, *G*c’ and *H*). Inhibition of Bmp2 by Noggin followed by Tun treatment caused no significant change in the expression of Chop (15.7 ± 2.9%; Fig. 4, *G*d’’ and *H*) as compared with Tun treatment alone (11.9 ± 1.1%; Fig. 4, *G*d’ and *H*).

These results are consistent with our previous observation (Fig. 3) that Tbx20 is located upstream of Bmp2 in imparting protection against ER stress by increasing cardiomyocyte proliferation. These data also indicate the fact that exogenous administration of Bmp2 can impart protection during increased ER stress by increasing cardiomyocyte proliferation by a positive feed-forward mechanism.

### Prolonged Tun-induced ER stress in adult heart is accompanied by altered cardiac functions, increased cell size, and collagen deposition *in vivo*

In order to validate the direct protective effect imparted by Tbx20–Bmp2 signaling from ER stress–induced apoptosis *in vivo*, we administered Swiss Albino mice with 1 mg/kg body weight (BW) Tun intraperitoneal injections for 8 h and 2 days. The two time points were chosen to depict ER stress induction for short interval (8 h) and long interval (2 days) in order to corroborate the *in vitro* results.

**Figure 5:**
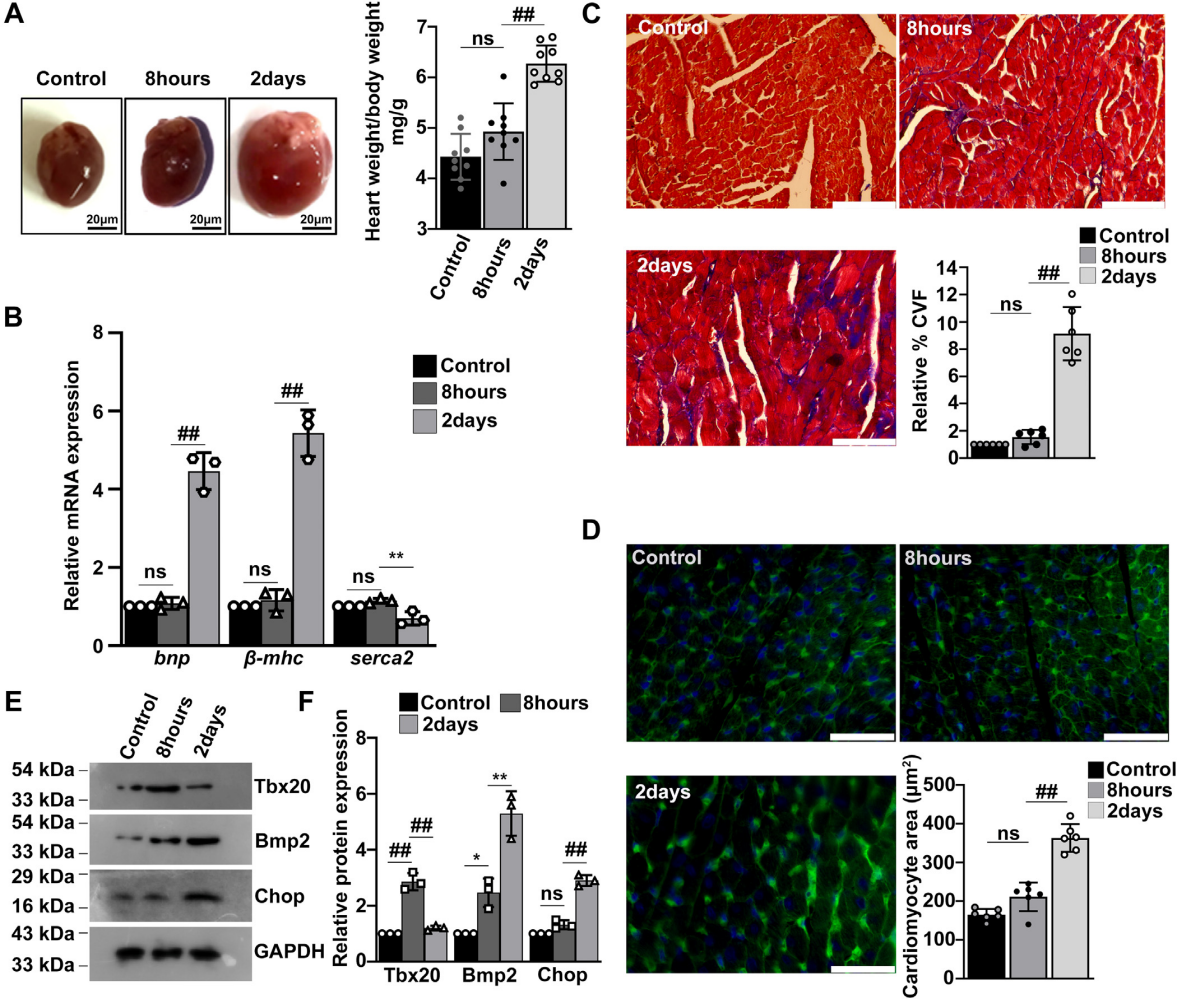
**Prolonged endoplasmic reticulum (ER) stress in adult murine heart results in altered cardiac function with increased cardiomyocyte size and collagen deposition in adult murine heart.***A*, the heart weight to body weight ratios indicative of cardiac hypertrophy is increased significantly during prolonged (2 days) ER stress as compared with ER stress induction for short duration (8 h) and control group. The change in heart weight to body weight ratio between 8 h ER stress and control group was however negligible (n = 9). Scale bar represents 20 μm. *B*, quantitative RT–PCR (qRT–PCR) analysis showed that prolonged (2 days) ER stress induction resulted in significant increase in the expression of cardiac function test markers *bnp* and *β-mhc* and significant decrease in the expression of *serca2* as compared with 8 h ER stress induction group. *C*, prolonged (2 days) ER stress results in increased collagen deposition indicative of cardiac fibrosis as shown in Masson’s trichrome-stained adult heart sections as compared with 8 h ER stress group and control group (n = 6). *D*, in prolonged (2 days) ER stress–induced adult mice, cardiomyocyte cell size is increased marked by wheat-germ agglutinin (WGA) staining (*green*) in comparison to 8 h ER stress group and control group, respectively (n = 6). *E*, Western blot analysis of Tbx20 showed a significant increase in its expression in 8 h Tun-treated group as compared with control. The expression of Tbx20 later decreased significantly in the 2-day group. The expression of Bmp2 increased significantly during 2 days as compared with 8 h Tun-treated group. The expression of apoptotic marker Chop increased significantly in the 2-day group as compared with 8 h and control group. *F*, quantitative representation by ImageJ software of the proteins using three biological replicates from *E*. Scale bar represents 50 μm. Error bars represent SD from at least three independent biological replicates. Statistical significance was calculated by one-way ANOVA. ns, *p*: nonsignificant, ∗*p* < 0.05, ∗∗*p* < 0.005, ∗∗∗*p* < 0.0005, ^##^*p* < 0.0001; n ≥ 3 independent experiments. Tbx20, T-box transcription factor 20.

Morphological abnormalities like hypertrophy (increased cardiomyocyte size) and fibrosis (increased collagen deposition) were detected upon prolonging ER stress in heart as compared with ER stress induction for short interval as well as control heart samples. Prolonged ER stress resulted in significant increase in heart weight (HW)/BW ratio (6.27 ± 0.36 mg/g) as compared with ER stress induction for short interval (4.92 ± 0.55 mg/g) and control group (4.42 ± 0.45 mg/g) (Fig. 5*A*).

Cardiac function in mice following ER stress induction was checked by assessing the change in expression of brain natriuretic peptide (*bnp*), β myosin heavy chain (*β-mhc*), and sarcoendoplasmic reticulum calcium ATPase 2 (*serca2*). Previous studies reported *bnp* and *β-mhc* as a diagnostic biomarker for cardiac dysfunction (30, 31). Cardiac *serca2* was also previously reported as therapeutic targets for heart failure (32). Our study showed an increase in the expression of *bnp* during 2 day Tun treatment (4.46 ± 0.47-fold) as compared with 8 h Tun treatment (1.08 ± 0.15-fold) and control mice (Fig. 5*B*). The expression of *β-mhc* was also increased during 2 day treatment as compared with 8 h and control mice. The expression of *serca2*, another therapeutic target for cardiomyopathy, was decreased significantly (0.7 ± 0.17-fold) during 2 day of ER stress induction in mice as compared with 8 h (1.15 ± 0.06-fold) and control mice (Fig. 5*B*).

Masson’s trichrome-stained adult heart sections revealed increased fibrotic regions marked by increased collagen deposition in prolonged ER stress (2 days)–induced heart tissue as compared with ER stress induced for short interval (8 h) heart tissue and control group (Fig. 5*C*). However, the presence of fibrotic regions in the 8 h ER stress–induced heart tissue was nonsignificant in comparison to control mice. Cardiomyocyte cell size was increased significantly in 2 day Tun-treated hearts (363.3 ± 35.74 μm^2^) as compared with 8 h ER stress–induced hearts (211.3 ± 36.89 μm^2^) and control group (165 ± 14.93 μm^2^) as indicated by wheat germ agglutinin staining (Fig. 5*D*). The increase in cell size was however nonsignificant between 8 h ER stress–induced heart and control group. These data show that prolonged ER stress (2 days) is accompanied by increased cardiomyocyte hypertrophy and fibrosis as compared with ER stress interval for shorter interval of time (8 h).

Western blot analysis revealed an increase in the expression of Tbx20 (2.86 ± 0.3-fold) during 8 h ER stress induction as compared with control group. However, a prolonged ER stress (2 days) resulted in significant decrease (1.2 ± 0.08-fold) in the expression of Tbx20 (Fig. 5, *E* and *F*). The expression of Bmp2 also increased during 8 h (2.46 ± 0.5-fold) ER stress induction as compared with control group. However, its expression increased significantly during 2 day ER stress induction group (5.3 ± 0.7-fold; Fig. 5, *E* and *F*). Decrease in the expression of Tbx20 during prolonged ER stress was accompanied by increased expression of apoptosis inducer Chop (2.9 ± 0.19-fold; Fig. 5, *E* and *F*) as compared with 8 h (1.32 ± 0.15-fold) and control group.

Together, these data suggest that ER stress induction for short interval (8 h), where the expression of Tbx20 is increased, resulted in no significant change in the expression of markers of cardiac function, hypertrophy, and fibrosis. However, prolonging the ER stress (2 days) resulted in significant decrease in the expression of Tbx20 with concomitant alteration in cardiac function of mice.

Next, we examined whether Tbx20 imparts its protective function during ER stress–mediated cardiomyopathy in rats also. Prolonged ER stress induction for 2 days in rats resulted in significant increase in HW to BW ratio (6.1 ± 0.1-fold) as compared with ER stress induction for 8 h (5.0 ± 0.05-fold) and control group (4.9 ± 0.16-fold; Fig. S3*A*). Western blot of Tbx20 showed an increase in its expression in 8 h Tun treatment group (2.7 ± 0.1-fold) as compared with control group. However, upon prolonging the ER stress to 2 days, the expression of Tbx20 was decreased significantly (0.6 ± 0.2-fold; Fig. S3, *B* and *C*). The expression of Bmp2 was also increased during 8 h (3.5 ± 0.34-fold) as compared with control. The expression of Bmp2 however increased drastically in 2 day Tun-treated group (8.6 ± 0.7-fold; Fig. S3, *B* and *C*). Decrease in the expression of Tbx20 in the 2-day group resulted in concomitant increase in the expression of apoptotic marker Chop (4.7 ± 0.5-fold) as compared with 8 h group (1.5 ± 0.09-fold; Fig. S3, *B* and *C*).

Cardiac function study of the rats revealed a significant increase in the expression of *bnp* and *β-mhc* in the 2-day group as compared with 8 h and control group. The change in the expression of *bnp* and *β-mhc* between 8 h and control group was nonsignificant (Fig. S3*D*). The expression of *serca2* decreased in the 2 day group as compared with the 8 h and control group (Fig. S3*D*). ECG measurement of all the animals of each group was recorded. ECG recordings of the 2 day ER stress group resulted in significant increase in QT interval (0.093 ± 0.01 s) as compared with 8 h (0.077 ± 0.01 s) and control group (0.072 ± 0.01 s; Fig. S3, *E* and *F*). The RR interval of the 2 day group showed a significant decrease (0.19 ± 0.01 s) as compared with 8 h (0.24 ± 0.01 s) and control (0.25 ± 0.01 s) group (Fig. S3, *E* and *G*). The 2 day group also showed an elevation of the ST segment as compared with 8 h and control group (Fig. S3*E*).

Therefore, all these observations highlight the importance of Tbx20 in maintaining proper cardiac function. When the expression of Tbx20 is decreased during prolonged ER stress (2 days), the cardiac function is impaired resulting in progression of cardiomyopathy because of ER stress.

### ER stress–induced upregulation of Tbx20 activity is beneficial for cardiomyocyte viability and maintenance of cardiomyocyte homeostasis by regulating proliferation and apoptosis in adult murine heart

**Figure 6:**
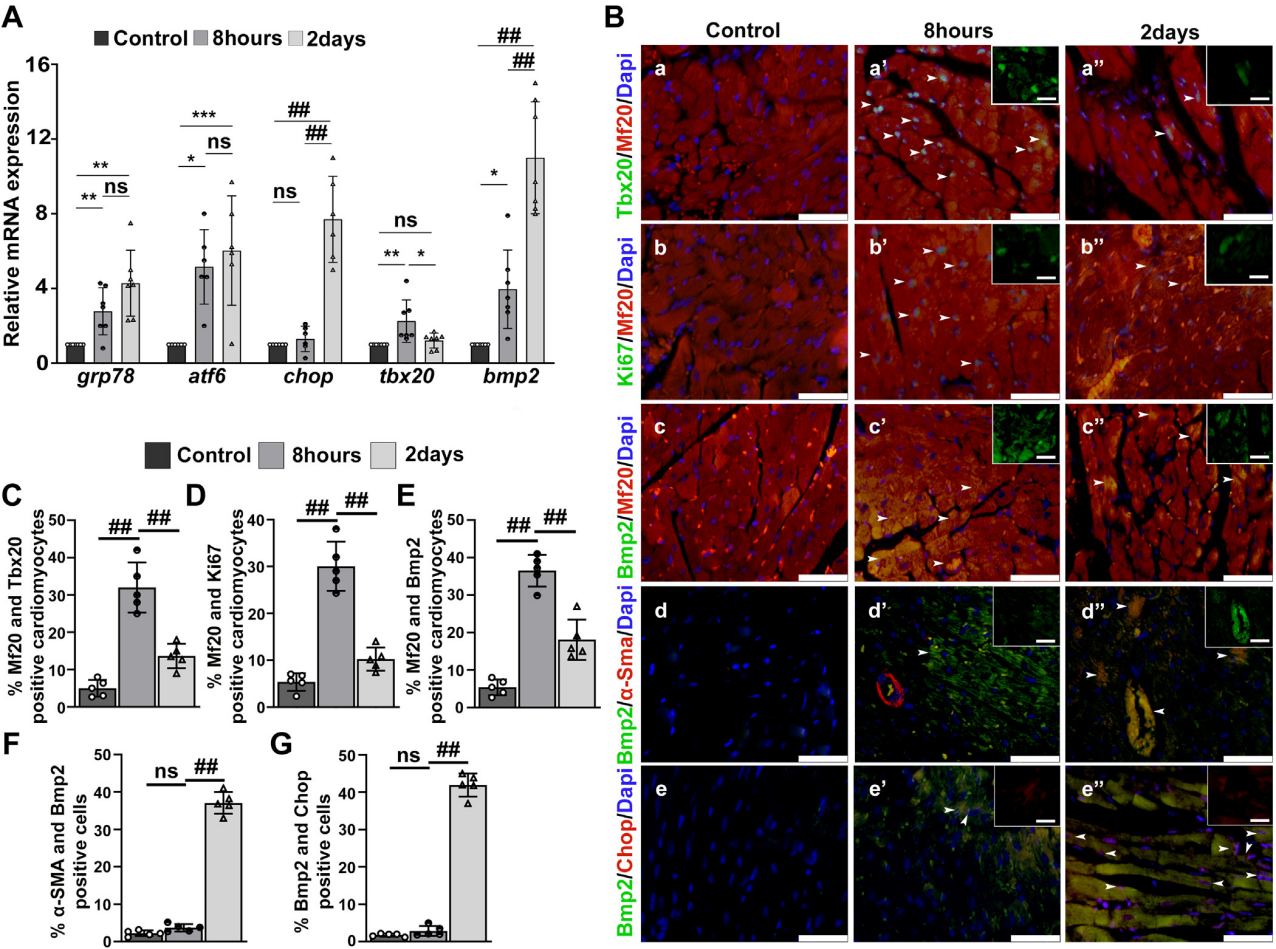
**Endoplasmic reticulum (ER) stress–mediated upregulation of Tbx20–Bmp2 signaling results in increased proliferation and limits apoptosis in adult murine hearts.***A*, ER stress induction for short duration (8 h) resulted in increase in the expression of ER stress markers *grp78* and *atf6* compared with control group as determined by quantitative RT–PCR (qRT–PCR). However, the change in expression of *atf6* and *grp78* between 8 h and prolonged ER stress (2 days) group was found to be negligible. The expression of apoptosis inducer *chop* increased significantly in prolonged ER stress (2 days) group as compared with 8 h and control groups. The change in expression of *chop* between 8 h and control group was nonsignificant. The expression of *tbx20* and *bmp2* increased during ER stress induction for short duration (8 h) as compared with control; however, the expression of *bmp2* increased significantly in the 2 day ER stress–induced group. *B*, ER stress induction for short duration (8 h) resulted in cardiomyocyte-specific increase in the expression of Tbx20 (a’) marked by Tbx20-positive nuclei (*green*) colabeled with cardiomyocyte-specific Mf20 (*red*) compared with control (a). However, a prolonged ER stress resulted in decrease in the expression of Tbx20 (a’’). The expression of proliferation marker Ki67 increased significantly as marked by increased Ki67-positive nuclei (b’; *green*) colabeled with cardiomyocyte-specific Mf20 (*red*) during 8 h of ER stress induction compared with control (b). Prolonged ER stress (2 days) resulted in decreased expression of Ki67 (b’’) compared with 8 h ER stress induction group. Cardiomyocyte-specific expression of Bmp2 was increased in 8 h ER stress (c’) as compared with control (c). However, its expression later decreased during 2 day ER stress (c’’) induction group. Bmp2 was also shown to colocalize with α-SMA with increased expression during prolonged ER stress (d’’) condition as compared with 8 h ER stress (d’) and control (d) groups. Increase in Bmp2 expression was accompanied by increased expression of Chop during prolonged ER stress (e’’). *C*–*G*, quantitative representation of panels in *B*. Scale bar of *main images* represents 50 μm. Scale bar of *inset* represents 20 μm. Statistical significance was calculated by one-way ANOVA. Error bars represent SD from at least three independent biological replicates (n ≥ 3); ns, *p*: nonsignificant, ∗*p* < 0.05, ∗∗*p* < 0.005, ∗∗∗*p* < 0.0005, ^##^*p* < 0.0001. Bmp2, bone morphogenetic protein 2; Tbx20, T-box transcription factor 20; α-SMA, alpha-smooth muscle actin.

Next, the status of Tbx20 activity and its function in Tun-treated adult murine hearts *in vivo* was examined. Similar results as that of protein levels were observed at transcript level. The establishment of ER stress was accessed by checking the expression of ER stress markers *grp78* and *atf6* by quantitative RT–PCR (qRT–PCR). ER stress induction for short period (8 h) resulted in a significant increase in expression of *grp78* (2.8 ± 1.2-fold) as compared with control. Its expression also increased during prolonged ER stress (4.2 ± 2.6-fold) as compared with control; however, the increase was nonsignificant in comparison to 8 h ER stress group (Fig. 6*A*). The expression of another ER stress marker *atf6* was also increased during prolonged ER stress (6.0 ± 2.9-fold) as compared with 8 h ER stress treatment group (5.2 ± 1.9-fold); however, that change was nonsignificant. The expression of apoptosis inducer *chop* did not change significantly in the 8 h ER stress group (1.29 ± 0.6-fold) with respect to control group. However, its expression increased significantly in the 2 day ER stress group (7.7 ± 2.3-fold) (Fig. 6*A*). The expression of *tbx20* increased significantly during 8 h ER stress (2.3 ± 1.1-fold) with reference to control group. On the contrary, a prolonged ER stress resulted in significant decrease in the expression of *tbx20* (1.2 ± 0.4-fold) as compared with 8 h ER stress–induced (Fig. 6*A*) group. These results are consistent with the *in vitro* results. ER stress induction for a shorter interval leads to the upregulation of *tbx20*, which in turn accelerates the expression of protective ER gene *atf6*. Prolonging the ER stress eventually leads to decrease in the expression of *tbx20*. A nonsignificant increase in the expression of *atf6* in the prolonged ER stress group might be attributed to the fact that the heart is composed of heterogeneous population of cells as compared with pure cardiomyocyte population of H9c2 cells; hence, the expression of *atf6* might be regulated by factors other than *tbx20* in murine heart. Another interesting observation is the drastic increase (11 ± 2.9-fold) in the expression of *bmp2* in 2-day ER stress group with reference to 8 h ER stress group (3.96 ± 2.0-fold) as opposed to the *in vitro* data (Fig. 6*A*). The drastic increase of *bmp2* in the prolonged ER stress group may also be due to the heterogeneity of the adult mice heart.

The requirement for Tbx20 in adult cardiomyocyte homeostasis post ER stress induction *in vivo* was determined by immunostaining. Colocalization of Tbx20 and Mf20^+^ (cardiomyocyte-specific marker) revealed a significant increase in the expression of Tbx20 in the cardiomyocytes of 8 h group (32 ± 6.6%; Fig. 6, *B*a’ and *C*) with respect to control (5.02 ± 2.2%; Fig. 6, *B*a and *C*). However, its expression decreased in the 2 day group (13.64 ± 3.2%; Fig. 6, *B*a’’ and *C*) in comparison to 8 h group. The results correlated with the mRNA data. Increase in the expression of Tbx20 was accompanied with increased cardiomyocyte proliferation. Colocalization of Ki67 and Mf20^+^ showed an increase in cardiomyocyte proliferation marked by increased Ki67-positive nuclei in the 8 h group (30.06 ± 5.2%; Fig. 6, *B*b’ and *D*) compared with control group (5.36 ± 1.8%; Fig. 6, *B*b and *D*). However, it decreased in the 2 day group (10.24 ± 2.4%; Fig. 6, *B*b’’ and *D*), which is suggestive of decreased proliferation because of decrease of Tbx20. Bmp2 was shown to colocalize along with Mf20^+^, and its expression was increased significantly in 8 h group (Fig. 6, *B*c’ and *E*) with respect to control group (Fig. 6, *B*c and *E*). However, its expression later decreased in 2 day group (Fig. 6, *B*c’’ and *E*). In order to validate the drastic increase of *bmp2* mRNA, colocalization of Bmp2 with α-SMA (myofibroblast-specific marker) was performed. Bmp2 was shown to colocalize with alpha-smooth muscle actin (α-SMA) in the 2 day group, and its expression was increased significantly (37.08 ± 2.9%; Fig. 6, *B*d’’ and *F*) as compared with the 8 h group (3.7 ± 0.9%; Fig. 6, *B*d’ and *F*). This observation supports the notion that drastic increase in Bmp2 expression is attributed to cell types other than cardiomyocytes in adult mice heart *in vivo*. Drastic increase in Bmp2 expression was accompanied by increased expression of Chop in 2 day group (Fig. 6, *B*e’’ and *G*). The increase in the expression of Bmp2 and subsequent increase in the expression of Chop during prolonged ER stress may be due to increased inflammatory response and is discussed in detail in the Discussion section.

Taken together, these observations further strengthen our hypothesis that there is a fine balance between ER stress–induced survival and death. ER stress induction for short interval leads to upregulation of Tbx20, which eventually caused increased cardiomyocyte proliferation because of increased expression of Bmp2 and limits cardiomyocyte apoptosis. However, a prolonged ER stress abrogates the expression of Tbx20 resulting in decreased proliferation and increased cardiomyocyte apoptosis, which leads to disruption of the homeostasis eventually leading to cardiomyocyte death.

### Hyperglycemia-induced ER stress upregulates activity of Tbx20 with concomitant increase in cardiomyocyte proliferation

Diabetic heart disease accounts for almost 80% of deaths among the patients suffering from diabetes. The mechanisms that cause gradual cardiomyocyte apoptosis in chronic diabetes are multifactorial, but recent evidence suggest the involvement of the ER stress in the cardiac apoptosis in a streptozotocin-induced type 1 diabetic rat model and in hyperglycemia (33, 34). ER stress has been implicated to induce fibrosis and cardiomyocyte death or apoptosis in diabetic cardiomyopathy. Therefore, to validate our results in a disease model, we have chosen diabetic cardiomyopathy as our model system.

**Figure 7:**
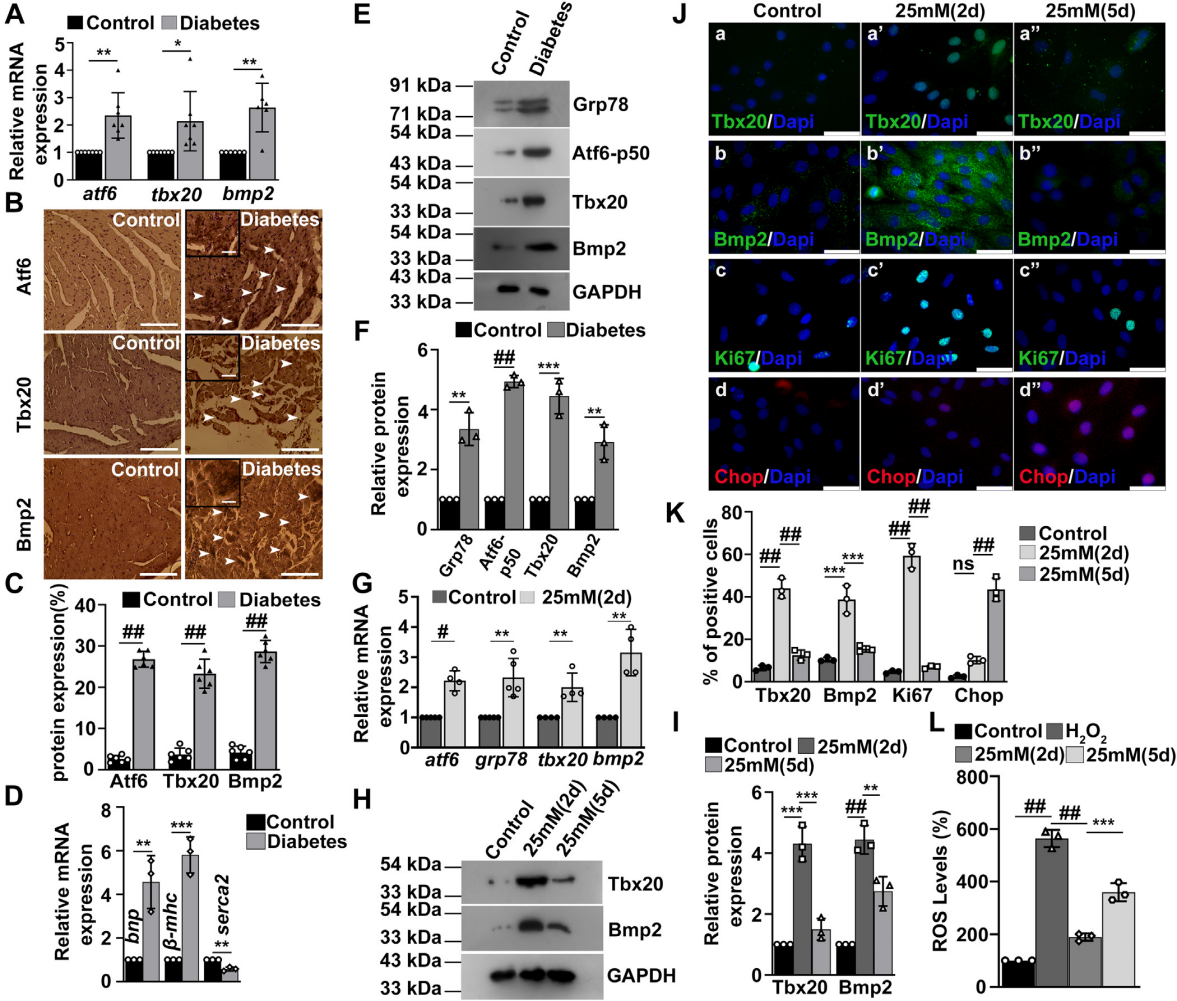
**Hyperglycemia-induced endoplasmic reticulum (ER) stress increases the activity of Tbx20–Bmp2 signaling axis with concomitant increase in proliferation and decrease in apoptosis.***A*, induction of diabetes in mice resulted in increased expression of *atf6*, *tbx20*, and *bmp2* as determined by quantitative RT–PCR (qRT–PCR) analysis. *B*, immunohistochemical analysis revealed increased expression of Atf6, Tbx20, and Bmp2 upon diabetes induction *in vivo*. Scale bar of *main images* represents 50 μm. Scale bar of *inset* represents 20 μm. *C*, quantitative representation of panel in *B*. Statistical significance was calculated by Student’s *t* test (n ≥ 6). *D*, qRT–PCR analysis showed an increase in the expression of *bnp* and *β-mhc* and decrease in the expression of *serca2* in the diabetes group as compared with control group. *E*, Western blot analysis showed an increase in the ER stress markers Grp78 and Atf6-p50 upon diabetes induction as compared with control. The expression of Tbx20 and Bmp2 is also increased during diabetes. *F*, quantitative representation by ImageJ software of the proteins using three biological replicates from *E*. *G*, qRT–PCR analysis showed an increase in the expression of *atf6*, *grp78*, *tbx20*, and *bmp2* upon hyperglycemia induction in cultured H9c2 cells. *H*, Western blot analysis showed an increase in the expression of Tbx20 and Bmp2 during hyperglycemia induced for 2 days (25 mM 2d). Prolonging the hyperglycemic stress for 5 days (25 mM 5d) resulted in decrease in the expression of Tbx20 and Bmp2. *I*, quantitative representation by ImageJ software of the proteins using three biological replicates from *H*. *J*, immunofluorescence staining showed an increase in the expression of Tbx20 (a’) and Bmp2 (b’) upon hyperglycemia induction for 2 days in comparison to respective controls (a and b). However, prolonging the hyperglycemia (5 days) resulted in decrease in their expression (a’’ and b’’). Increase in Tbx20 resulted in concomitant increase in cardiomyocyte proliferation marked by increased Ki67 (c’) as compared with control cells (c). However, prolonging the stress resulted in decrease in its expression (c’’). Prolonged hyperglycemic stress resulted in increased cardiomyocyte apoptosis marked by increased Chop (d’’) expression compared with hyperglycemic stress for 2 days (d’) and control. Scale bar represents 50 μm. *K*, quantitative representation of panels in *J*. *L*, reactive oxygen species (ROS) levels were increased upon prolonging the hyperglycemic stress for 5 days (25 mM 5d) as compared with 2 days (25 mM 2d) and control. Statistical significance was calculated by one-way ANOVA for three independent biological experiments (n = 3). Error bars represent SD from three independent biological replicates. ns, *p*: nonsignificant, ∗*p* < 0.05, ∗∗*p* < 0.005, ∗∗∗*p* < 0.0005, ^##^*p* < 0.0001. Atf6, activating transcription factor 6; Bmp2, bone morphogenetic protein 2; Tbx20, T-box transcription factor 20.

The establishment of ER stress during diabetes was validated by the expression of ER stress markers. The mRNA level of *atf6* was increased significantly (2.35 ± 0.8-fold) during diabetes as compared with control (Fig. 7*A*). The expression of *tbx20* and *bmp2* was also increased significantly during diabetes as compared with control (Fig. 7*A*). The protein expression of Atf6 was increased significantly during diabetes (25.12 ± 3.3%, Fig. 7, *B* and *C*) as compared with control (2.6 ± 0.8%). The expression of Tbx20 was also increased significantly during diabetes (23.28 ± 3.5%) as compared with control (3.68 ± 1.5%, Fig. 7, *B* and *C*). Bmp2 levels increased significantly in diabetes group (28.6 ± 2.6%) with reference to control group (4.16 ± 1.5%) (Fig. 7, *B* and *C*). Thus, diabetes augments ER stress with concomitant increase in the expression of Tbx20 and Bmp2. Diabetic cardiomyopathy was accompanied by altered cardiac function as marked by increased expression of *bnp* and *β-mhc* and decreased expression of *serca2* as compared with control group (Fig. 7*D*). Western blot analysis showed a significant increase in the expression of Grp78 (3.35 ± 0.5-fold; Fig. 7, *E* and *F*) as compared with control group. The expression of Atf6 also increased significantly (4.9 ± 0.2-fold; Fig. 7, *E* and *F*). Diabetic cardiomyopathy also resulted in increased expression of Tbx20 (4.4 ± 0.5-fold) and Bmp2 (2.9 ± 0.5-fold) as compared with control group (Fig. 7, *E* and *F*).

Similarly, the increased expression of ER stress genes *atf6* and *grp78* was observed in H9c2 cells with high concentration of glucose (25 mM) for 2 days. Increase in ER stress was accompanied by increase in the expression of *tbx20* (2.0 ± 0.4-fold) and *bmp2* (3.15 ± 0.7-fold) as compared with cells (Fig. 7*G*).

Western blot analysis revealed an increase in the expression of Tbx20 (4.3 ± 0.5-fold) and Bmp2 (4.4 ± 0.4-fold) when H9c2 cells were treated with higher glucose for 2 days. However, prolonging the ER stress for 5 days resulted in decreased expression of Tbx20 (1.4 ± 0.3-fold) and Bmp2 (2.7 ± 0.4-fold; Fig. 7, *H* and *I*).

The expression of Tbx20 increased up to 43.97 ± 4.5% (Fig. 7, *J*a’ and *K*) in the 2 day group as compared with control group (6.53 ± 1.2%; Fig. 7, *J*a and *K*). However, prolonging the hyperglycemic stress for 5 days resulted in significant decrease in the immunoreactivity of Tbx20 (12.57 ± 2.4%; Fig. 7, *J*a’’ and *K*) as compared with 2 days. The expression of Bmp2 also showed a similar increase in immunoreactivity in 2 day group (38.73 ± 6.6%; Fig. 7, *J*b’ and *K*) as compared with control (10.5 ± 1.1%; Fig. 7, *J*b and *K*) followed by decrease in 5 days (15.47 ± 1.4%; Fig. 7, *J*b’’ and *K*). Increase in Tbx20 was accompanied by increased nuclear immunoreactivity of Ki67 in the 2 day group (59.37 ± 5.7%; Fig. 7, *J*c’ and *K*) compared with control group (4.66 ± 0.6%; Fig. 7, *J*c and *K*). However, with decreased expression of Tbx20, the expression of Ki67 also decreased in the 5 day group (6.92 ± 0.8%; Fig. 7, *J*c’’ and *K*). Decrease in the activity of Tbx20 in 5 days was accompanied by increased apoptosis marked by augmented expression of Chop (57.97 ± 5.5%; Fig. 7, *J*d’’ and *K*) as compared with the 2 day group (10.23 ± 1.6%; Fig. 7, *J*d’ and *K*). Increase in the duration of hyperglycemic stress to 5 days in H9c2 cells resulted in increased ROS levels as compared with hyperglycemic stress induced for 2 days (Fig. 7*L*).

Thus, these observations further confirm that ER stress–mediated cardiomyopathy results in the upregulation of Tbx20 and Bmp2 with concomitant increase in cardiomyocyte proliferation. However, prolonging the stress eventually leads to decreased Tbx20 expression with concomitant decrease in proliferation and increase in apoptosis of cardiomyocytes.

## Discussion

The adult heart proliferates at a lower level strengthens the fact that cardiomyocyte can repair postinjury. Here, we show that ER stress–mediated activation of Tbx20 promotes cardiomyocyte proliferation and limits cardiomyocyte apoptosis by activating Bmp2–pSmad1/5/8 pathway and upregulating the expression of cardioprotective Atf6 arm of UPR. The balance between ER stress–mediated cardiomyocyte survival and ER stress–mediated cardiomyocyte apoptosis is a critical factor that directs the protective effect of Tbx20, and it must be taken into account while considering therapeutic approaches to ER stress–mediated cardiomyopathies. Tbx20 overexpressing cardiomyocyte or cardiomyocyte-specific induction of Bmp2 could provide protection even during prolonged ER stress and requires further studies. Thus, studies on induction of cardiogenic gene Tbx20 or upregulation of the Tbx20–Bmp2–pSmad1/5/8 pathway in adult cardiomyocytes can protect them from ER stress–mediated cardiomyopathy and promote their regeneration.

**Figure 8:**
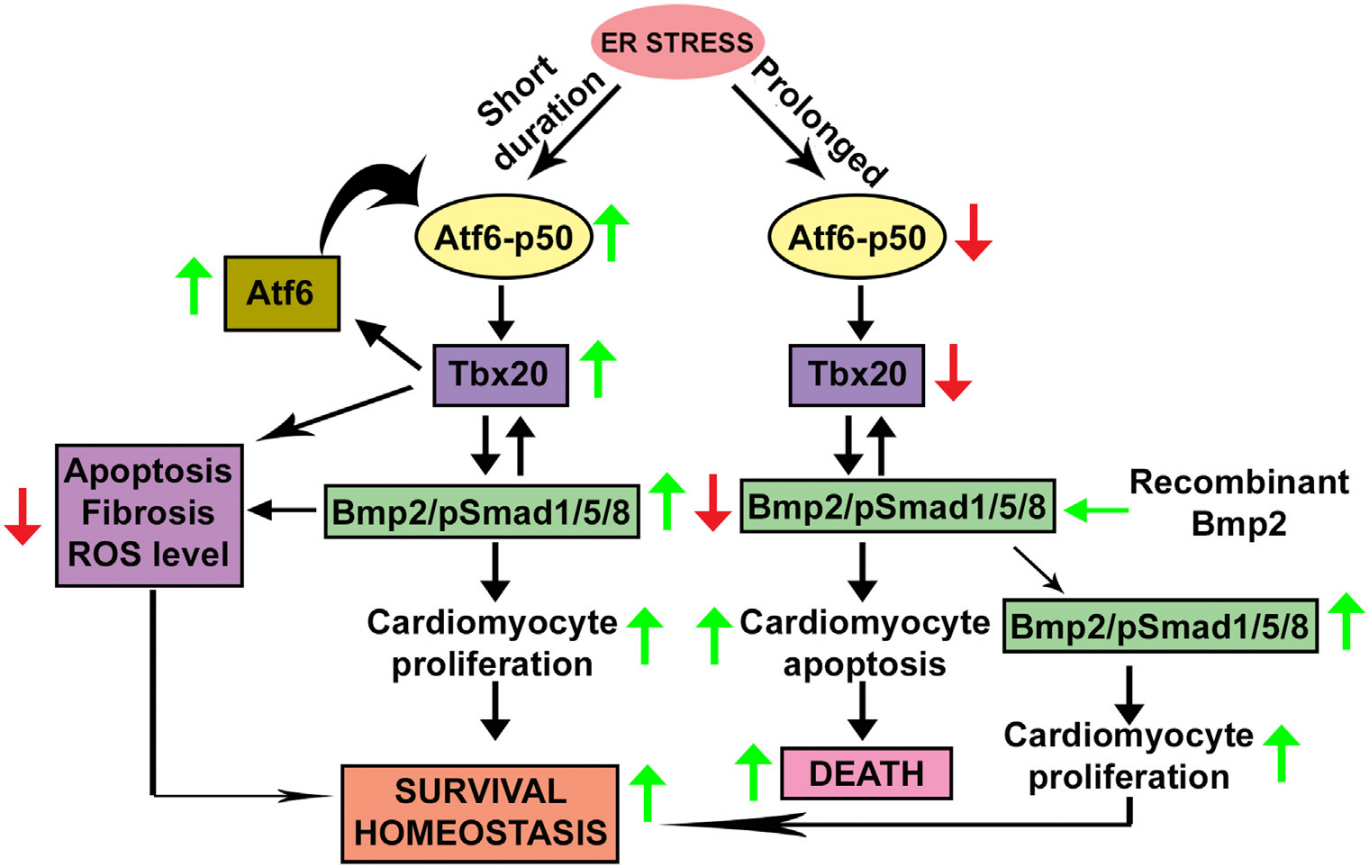
**Model for endoplasmic reticulum (ER) stress–mediated upregulation of T-box transcription factor 20 (Tbx20) resulting in increased cardiomyocyte survival.** ER stress induction for short interval results in upregulation of activating transcription factor 6 (Atf6) pathway of unfolded protein response (UPR). Full-length Atf6 is cleaved (Atf6-p50), and it translocates to the nucleus where it increases the transcription of Tbx20. Increase in the level of Tbx20 results in upregulation of its downstream signaling cascade consisting of bone morphogenetic protein 2 (Bmp2)–pSmad1/5/8. Increased expression of Bmp2–pSmad1/5/8 further results in increased cardiomyocyte proliferation, thereby restoring homeostasis and increasing the survival of cardiomyocyte post ER stress induction. Tbx20 also helps in restoration of homeostasis by decreasing apoptosis, fibrosis, and reactive oxygen levels (ROS) levels during ER stress. Increased expression of Tbx20 during ER stress also helps to maintain the total pool of Atf6 during ER stress. A prolonged ER stress however results in decreased expression of cleaved Atf6 (Atf6-p50). This in turn results in decrease in the expression of Tbx20, and it downstreams Bmp2–pSmad1/5/8 signaling molecules resulting in decreased cardiomyocyte proliferation and increased cardiomyocyte apoptosis. Ectopic administration of recombinant Bmp2 protein results in upregulation of Bmp2, which in turn upregulates Tbx20 in a feed-forward mechanism, thereby restoring homeostasis by increasing cardiomyocyte proliferation.

The adult mammalian heart possesses a little regenerative capacity, which is insufficient to compensate for the loss of cardiomyocyte because of pathophysiological conditions. Thus, triggering on the proliferative capacity of the pre-existing cardiomyocytes of adult heart represents promising strategy for restoration of cardiac homeostasis postinjury (35). Tbx20 represses cell cycle inhibitory genes *p21*, *meis1*, *and btg2*, thereby promoting adult cardiomyocyte proliferation post myocardial infarction suggesting a critical mediator for cardiomyocyte proliferation postinjury (36). Previous studies have shown that Bmp2 protects cardiomyocytes from ischemia/reperfusion injury *via* upregulating the Smad1 pathway, which in turn inhibits apoptosis (37). The fact that Tbx20 and Bmp2 are essential factors for cardiomyocyte proliferation post ER stress–mediated injury was unknown so far. Our study has shown a regulatory mechanism whereby ER stress–mediated upregulation of Tbx20 leads to cardioprotection and restoration of cardiac homeostasis in H9c2 cardiomyocytes and adult mice heart by augmenting cardiomyocyte proliferation and limiting cardiomyocyte apoptosis *via* upregulating the Bmp2–pSmad1/5/8 pathway (Fig. 8). However, prolonging the extent of ER stress eventually results in reversal of this phenomenon with decreased Tbx20–Bmp2 expression, decreased cardiomyocyte proliferation, and increased cardiomyocyte apoptosis. Our study for the first time showed that Atf6 directly binds to the promoter of *tbx20* gene during ER stress condition, thereby increasing its expression. Knockdown of Tbx20 followed by ER stress induction was shown to decrease Bmp2 signaling with concomitant decrease in cardiomyocyte proliferation and increase in cardiomyocyte apoptosis. Previous studies have shown that overexpression of Tbx20 results in reduction of fibrotic scars (36). Knockout of Tbx20 on the other hand was shown to cause extensive fibrosis within a short period (10). Bmp2 was also shown to decrease renal interstitial fibrosis and liver fibrosis (38, 39). Our study showed the mode of action of Tbx20 during ER stress. First, ER stress–mediated increase in the expression of Tbx20 leads to the upregulation of Bmp2–pSmad1/5/8, which in turn increases cardiomyocyte proliferation. Second, Tbx20 was shown to decrease the expression of both apoptotic and fibrotic markers during ER stress, thereby restoring homeostasis. ER stress–mediated induction of Tbx20 was also shown to restrict the levels of ROS generation because of ER stress. The study also showed that Bmp2 acts downstream of Tbx20 in imparting protection against ER stress–induced cardiomyopathy.

Previous studies have shown the use of RecBmp2 protein for ectopic overexpression of Bmp2 (22, 38, 40). Investigation of the relationship among this pathway by administration of RecBmp2 protein following ER stress induction (50 μg/ml Tun) revealed that Bmp2 can exert its synergistic effect on Tbx20 by increasing the expression of Tbx20 with concomitant increase in cardiomyocyte proliferation. However, Noggin treatment followed by ER stress induction (10 μg/ml Tun) caused no change in the expression of Tbx20 as compared with ER stress induction alone, thus proving the presence of a feed-forward loop mechanism in this pathway. We have also reported an increase in the activity of Tbx20 and Bmp2 during hyperglycemia *in vitro* and diabetic cardiomyopathy *in vivo* with concomitant increase in cardiomyocyte proliferation. Together, these data support a regulatory mechanism whereby Tbx20–Bmp2 signaling imparts its protective role in order to restore cardiomyocyte homeostasis during ER stress and during hyperglycemic condition.

A disruption in the protein assembly machinery results in the generation of ER stress with subsequent upregulation of the UPR. There is increasing evidence suggesting that the balance of protective UPR and ER stress–mediated apoptosis regulates the progression of cardiovascular diseases. Atf6 has mostly been considered as a protective molecule during ER stress. Hence, in our study, we have mainly focused on the Atf6 arm of UPR. Atf6 overexpression protected heart from ischemia/reperfusion injury by improving left ventricle–developed pressure, reducing expression of apoptotic markers, and ameliorating infarct size (41). Constitutive expression of Atf6 was shown to improve cardiac function during ER stress–mediated cardiomyocyte apoptosis postinjury and during diabetes mellitus (42). Atf6 exerts its cardioprotective effect by augmenting the expression of SERCA2a, antioxidants catalase (Cat), peroxiredoxin 5 (Prdx5), and VCP-interacting membrane protein (Vimp) (43, 44). Here, we provide evidence that Atf6, a cardioprotective molecule of the UPR, is responsible for increase in the expression of Tbx20 during ER stress. Our study showed the direct DNA-binding ability of Atf6 to the promoter region of *tbx20*, thereby inducing its expression during ER stress. Our results were further corroborated by the use of Atf6 inhibitor AEBSF. We have shown by inhibiting the cleavage and nuclear translocation of active form of Atf6 (Atf6-p50) with AEBSF that it is indeed responsible for the increase in the expression of Tbx20 during ER stress.

Our study showed that the protective effect of Tbx20 during ER stress is mediated by Atf6 arm of the UPR signaling. However, mild ER stress also results in upregulation of the other two arms of UPR signaling pathway IRE1α and PERK. PERK pathway was shown to activate cytoprotective gene expression pathway, thus promoting stress-resistant state (45). PERK knockout mice in response to transverse aortic constriction was shown to display altered cardiac function and enhanced cardiac apoptosis (46). XBP1, which is a downstream molecule of IRE1α arm, was to increase vascular endothelial growth factor A–mediated angiogenesis in response to ER stress (47). Despite having cardioprotective effects, these two pathways can induce the transcription of apoptotic molecules that results in detrimental consequences resulting in cardiomyopathy. PERK pathway results in the upregulation of Chop and p53-upregulated modulator of apoptosis (Puma), which induces cardiomyocyte apoptosis resulting in cardiomyopathy (48). On the other hand, IRE1α results in the upregulation of apoptosis signal-regulating kinase 1 (ASK1), which is a critical molecule in eliciting cardiomyocyte death induced by ER stress (49). Thus, these two pathways need to be studied further to decipher whether they could influence cell survival during ER stress.

Our study reported that knockdown of Tbx20 followed by Tun treatment resulted in decreased expression of both total and cleaved form of Atf6. ChIP analysis further revealed direct DNA binding of Tbx20 to the promoter of *atf6* gene during ER stress, thereby showing that Tbx20 helps in maintaining the pool of total Atf6 during ER stress. Therefore, our study has showed the existence of a feedback mechanism between Tbx20 and Atf6 during ER stress. Tbx20 helps to maintain the pool of Atf6 during ER stress. On the other hand, during ER stress, Atf6 is cleaved, and it translocates to the nucleus where it drives the transcription of *tbx20* gene.

Cardiomyocyte-specific deletion of Tbx20 results in embryonic lethality as it is required for the regulation of genes involved in fetal cardiomyocyte proliferation (26). Loss of function of Tbx20 causes double outlet right ventricle and familial tetralogy of fallot (50, 51). On the contrary, overexpression of Tbx20 promotes adult cardiomyocyte proliferation post myocardial infarction (36), thus highlighting its importance for proper heart development and function. Our study reported an increase in the expression of Tbx20 during 8 h of ER stress induction. However, prolonging the ER stress to 2 days resulted in significant downregulation in its expression in both mice and rat. ECG analysis revealed an increase in the QT interval in 2 day group as compared with control and 8 h ER stress induction group. On the other hand, RR interval was significantly decreased in the 2 day treatment group. ST segment elevation was also observed in the 2 day group. QT prolongation was shown to cause malignant arrythmia and sudden cardiac death (52). On the other hand, shortening of the RR interval is indicative of increased heart rate (53). ST segment elevation was shown to be associated with left ventricular hypertrophy and acute myocardial infarction (54). All these parameters are established markers of cardiac function analysis. Since our study reported an alteration in these three parameters in the 2 day treatment group where the expression of Tbx20 is decreased in comparison to 8 h and control group, hence we can conclude that Tbx20 is indeed required for maintaining proper cardiac function during ER stress induction for short duration. Prolonging the ER stress eventually results in decreased expression of Tbx20 with concomitant alteration of cardiac function.

Our study reported a significant increase in the activity of Bmp2 during prolonged ER stress (2 days) in adult murine hearts. The increase in the expression of Bmp2 may be attributed to the fact that the adult murine heart is composed of multiple cell types as opposed to pure cardiomyocyte culture of H9c2 cells. The increase in Bmp2 expression may be due to the effect of ER stress on other cell types of adult heart. Bmp2 is expressed in multiple cell types of adult murine heart (55, 56). Colocalization studies have shown that Bmp2 colocalizes with α-SMA (myofibroblast marker) in the ER stress (2 days) group with increased expression as compared with ER stress (8 h), thus strengthening our plausible explanation. Sustained ER stress was shown to recruit tumor necrosis factor (TNF) receptor–associated factor 2 (TRAF2) and ASK-1, which causes subsequent activation of JNK and NF-κβ and production of proinflammatory cytokines like interleukin-1, interleukin-6, and TNF-α (57). Proinflammatory stimulus in turn regulates the activity of Bmp2. TNF-α and ROS generation increases the activity of Bmp2 (58). Bmp2 in turn induces proinflammatory endothelial phenotype. Thus, sudden increase in Bmp2 expression during prolonged ER stress may be attributed to other cell types of murine heart and because of increased inflammatory response. Since in our study the level of Bmp2 is already increased, hence we can conclude that *in vivo*, upon prolonging the ER stress, Tbx20-independent Bmp2 function is overriding the Tbx20-dependent protective function of Bmp2. Therefore, prolonging or maintaining the expression of Tbx20 for longer duration during ER stress could result in restoration of normal cardiac functions even during prolonged ER stress–mediated cardiomyopathy.

The ER is an organelle responsible for folding of proinsulin, and ER stress is implicated with the pathogenesis of diabetes mellitus. Hyperglycemia, a causative factor of diabetes mellitus, disrupts ER homeostasis resulting in development of ER stress (34). In order to replicate our observations in a disease model, we looked in the expression of Tbx20 during hyperglycemia *in vitro* and diabetic cardiomyopathy *in vivo*. Chronic hyperglycemia contributes to β-cell dysfunction by downregulating the expression of Atf6α/Ire1α, thus contributing to loss of homeostasis (59). Our study showed an increase in the expression of ER stress markers Atf6 and Grp78 upon induction of diabetes. The expression of Tbx20 and Bmp2 also increased with concomitant increase in cardiomyocyte proliferation upon induction of hyperglycemia. Prolonged hyperglycemia caused augmentation of ER stress with concomitant decrease of Tbx20–Bmp2 signaling, decrease in cardiomyocyte proliferation, and increase in cardiomyocyte apoptosis. Thus, Tbx20–Bmp2 signaling imparts protection against hyperglycemia by augmenting cardiomyocyte proliferation.

It was shown previously that increased ER stress is associated with increased ROS generation because of augmented disruption of disulphide bonds (19, 20). Our study reported a gradual increase in ROS generation with increase in the intensity of ER stress. However, when the expression of Tbx20–Bmp2 axis is decreased at a Tun concentration of 50 μg/ml, the ROS generation was increased drastically, and it almost correlated with positive control. Increased expression of Tbx20 was reported to result in decreased ROS generation in cardiomyocytes (21). Our study showed that knockdown of Tbx20 followed by ER stress induction resulted in significant increase in ROS generation as compared with ER stress induction alone, thus highlighting the role of Tbx20 in limiting ROS generation. Prolonged hyperglycemic stress where the expression of Tbx20 was decreased resulted in significant increase in ROS generation as compared with hyperglycemic stress induced for short interval of time where the expression of Tbx20 was high. Therefore, our study showed that ROS generation increases gradually with increase in ER stress. However, when the expression of Tbx20 is decreased during ER stress or during hyperglycemia, the ROS levels were increased significantly and was almost equal to that of positive control, thus corroborating the role of Tbx20 in limiting ROS generation during ER stress.

In conclusion, it is inferred from our study that Tbx20–Bmp2 signaling acts during ER stress–mediated cardiomyopathy by increasing cardiomyocyte proliferation and limiting cardiomyocyte apoptosis. We predict that overexpression of Tbx20 or cardiomyocyte-specific expression of Bmp2 signaling could be exploited as a novel therapeutic approach to confer protection against prolonged ER stress and shift the balance toward prosurvival to restore cardiac homeostasis during ER stress–induced cardiomyopathy.

## Experimental procedures

### Induction of ER stress *in vivo*

ER stress was induced *in vivo* by intraperitoneally injecting adult male Swiss Albino mice (8 weeks old) with Tun (catalog no.: T7765; Sigma–Aldrich) at a final concentration of 1 mg/kg BW diluted in sterile 150 mM dextrose (60), and the mice heart was harvested at 8 h and 2 days by euthanization using carbon dioxide, followed by cervical dislocation. The control mice were injected an equal volume of 150 mM dextrose containing 1% dimethyl sulfoxide. In adult male Wister rats, ER stress was induced by intraperitoneal injection of 1 mg/kg BW Tun diluted in sterile 150 mM dextrose (61). The rat hearts were harvested at 8 h and 2 days. All the animals were maintained as per Control and Supervision of Experiments on Laboratory Animals (CPCSEA) guidelines. All the animals were fed with normal chow diet and water *ad libitum*. All the experiments with animals were approved by the Institutional Animal Ethics Committee (IAEC), Jadavpur University (Ref no.: AEC/PHARM/1701/05/2017 dated November 12, 2020).

### Induction of diabetes *in vivo*

Male Swiss Albino mice (8 weeks old) was intraperitoneally injected with 150 mg/kg BW alloxan dissolved in 0.9% saline to induce diabetes (62). The control mice were treated with equal volume of 0.9% saline. Mice with blood glucose levels >200 mg/dl were maintained for up to 2 weeks for experimental purpose (63, 64). All the animals were maintained as per CPCSEA guidelines. All the animals were fed with normal chow diet and water *ad libitum*. All the experimental procedures were approved by the Institutional Ethical Committee, Presidency University (Registration PU/IAEC/SC/39), registered under “Committee for the purpose of CPCSEA, Ministry of Environment and forests, Govt. of India.

### Cell cultures and treatments

H9c2 cells were cultured in Dulbecco's modified Eagle's medium (Gibco) supplemented with 10% fetal bovine serum (Himedia), 100 units/ml penicillin G sodium, and 100 μg/ml streptomycin sulfate (catalog no.: 15140122; Gibco) in the presence of 5% CO_2_ at 37 °C. Upon reaching 60 to 70% confluency, the cells were starved for 6 h and treated with different concentration of Tun (2, 5, 10, 20, and 50 μg/ml) (65, 66) in order to induce ER stress. The cells were harvested after 24 h and used for further analysis. H9c2 cells were treated with different concentrations of DTT (catalog no.: D9779; Sigma–Aldrich) (1, 3, 5, and 10 mM) and Tg (catalog no.: T9033; Sigma–Aldrich) (1.5, 2, 6, and 10 μM) for 24 h to induce ER stress (67, 68).

For induction of hyperglycemia *in vitro*, the H9c2 cells were starved overnight in serum-free glucose free media prior to treatment. The cells were supplemented with 25 mM glucose and 5 mM glucose serving as hyperglycemic and control conditions, respectively. The culture media were replenished with respective media every alternative day. The H9c2 cells were harvested at 2 and 5 days and used for further analysis.

### 3-[4,5-Dimethylthiazol-2-yl]-2,5 diphenyl tetrazolium bromide cell viability assay

3-[4,5-Dimethylthiazol-2-yl]-2,5 diphenyl tetrazolium bromide assay was performed to monitor cell viability upon treatment of Tun for different time points. Cultured H9c2 cells were seeded in 96-well plates and treated with different concentrations of Tun (2, 5, 10, 20, and 50 μg/ml) for 24 h to induce ER stress. About 5 mg/ml of 3-[4,5-dimethylthiazol-2-yl]-2,5 diphenyl tetrazolium bromide (catalog no.: TC191; Himedia) stock solution was diluted in a ratio of 1:10 in 1× PBS. About 40 μl of diluted stock solution was added to each well. The cells were then incubated for 3 h in 5% CO_2_ at 37 °C. The solution was removed from each well, and 50 μl of extraction buffer (80% isopropanol, 20% Triton X-100, and 12 (N) HCl) was added to each well. The absorbance was measured at 570 nm.

### RNA interference and cell treatments

For siRNA transfection, H9c2 cells grown at 60 to 70% confluency were transfected with Tbx20 siRNA (assay ID: s164031; Ambion) at a final concentration of 100 nM (titrated for maximum downregulation) targeting the coding region of Tbx20 using Lipofectamine RNAiMAX (catalog no.: 13778-075; Invitrogen) reagent as per the manufacturer’s instructions after serum starvation overnight. The cells were maintained in transfection mix for 6 h in 37 °C in a 5% CO_2_ incubator and then cells were maintained in complete growth media (Dulbecco's modified Eagle's medium + 10% fetal bovine serum) for next 24 h. Control cells were transfected with scramble siRNA. About 24 h after siRNA transfection, the growth media were replenished with media containing 20 μg/ml Tun in order to induce ER stress and kept for another 24 h. The cells were harvested, and the cell lysate was used for Western blot analysis. Cultured cells treated with the aforementioned reagents were washed with 1× PBS and fixed with 4% paraformaldehyde for immunostaining purpose.

H9c2 cells were treated with 300 μM of water-soluble serine protease inhibitor (AEBSF) (catalog no.: A8456; Sigma–Aldrich) to inhibit the cleavage of membrane-bound Atf6 for 6 h followed by treatment of 20 μg/ml Tun for 24 h (69). The cells were harvested after 24 h, and the cell lysate was used for Western blot analysis.

Bmp2 recombinant protein (200 ng/ml, catalog no.: 355-BM; R&D Systems) and BMP inhibitor Noggin (200 ng/ml; catalog no.: 719-NG; R&D Systems) were used to overexpress and inhibit Bmp2, respectively. For overexpression of Bmp2, cultured H9c2 cardiomyocytes were first treated with 50 μg/ml Tun followed by treatment with Bmp2 recombinant protein for 24 h in parallel with vehicle control (0.1% bovine serum albumin [BSA] in 1× PBS). For inhibition of Bmp2, H9c2 cells were treated with Noggin for 24 h. This was followed by treatment with 10 μg/ml Tun to induce ER stress in parallel with vehicle control (1× PBS). The cells were harvested, and the cell lysate was used for Western blot analysis. Cultured cells treated with the aforementioned reagents were washed with 1× PBS and fixed with 4% paraformaldehyde for immunostaining purpose.

### Western blot analysis

Western blot was performed as described previously (22). Briefly, total protein was extracted from H9c2 cells from our different treatment conditions using protein lysis buffer (20 mM Tris–HCl, pH 7.5, 150 mM NaCl, 1 mM EDTA, 1 mM EGTA, 1% glycerol, 1% Nonidet P-40, 1 mM DTT, 100 mM NaF, 0.2 mM PMSF, and 1 mM Na_3_VO_4_) supplemented with protease inhibitor cocktail (catalog no.: GX-2811AR; Puregene) and phosphatase inhibitor cocktail (catalog no.: GX-1211AR; Puregene). A Bradford protein assay reagent (catalog no.: ML106; Himedia) was used to estimate the concentration of protein samples under different treatment conditions. About 60 to 100 μg of the protein extracts were fractionated using 7 to 12% SDS-PAGE under reducing conditions. The gels were then transferred onto polyvinylidene difluoride membrane (catalog no.: 1620177; Bio-Rad), and the membranes was blocked with 5% skimmed milk in Tris-buffered saline with Tween-20 for 1 h at room temperature. The membranes were then incubated with primary antibodies diluted in milk or BSA as per the manufacturer’s protocol at 4 °C overnight with constant shaking. The immunoblots were then incubated with horseradish peroxidase (HRP)–tagged secondary antibody and developed using Clarity Western ECL substrate (Luminol/enhancer solution and peroxide solution; catalog no.: 1610182; Bio-Rad).

### Immunostaining

The heart tissues sections were processed as described previously (22). Briefly following deparaffinization and rehydration in graded ethanol (100%, 95%, 75%, and 50%) and finally distilled water (two times), tissue sections were subjected to antigen retrieval in a microwave oven with citrate buffer (10 mM citric acid, 0.05% Tween-20, pH 6.0). Following antigen retrieval, tissue sections were incubated with blocking buffer (2% BSA, 0.1% Tween-20 in 1× PBS) for 1 h at room temperature. The sections were then incubated overnight at 4 °C with the respective primary antibodies as per experimental studies. Following incubation with the primary antibody, the sections were washed for three times with 1× PBS for 5 min each. The sections were then incubated with respective secondary antibody for 1 h at room temperature. Following antibody incubation, the sections were washed for three times with 1× PBS for 5 min each. The nuclei were counterstained with 4′,6-diamidino-2-phenylindole (catalog no.: D9542; Sigma–Aldrich) for 15 min at room temperature. The sections were washed for three times with 1× PBS for 5 min each and mounted in mounting media (20 mM Tris, pH 8.0, 0.5% N-propyl gallate, 90% glycerol). Images were taken by Leica DM2000 across different fields. For analysis of the tissue sections, at least three sections per mouse heart were used consisting a total of approximately 1000 to 1200 cardiomyocytes from experimental and littermate controls.

For *in vitro* studies, H9c2 cardiomyocytes cultured on coverslips were washed with PBS, followed by blocking (2% BSA and 0.1% Tween-20 in 1× PBS) and subsequent antibody incubation. Images were taken by Leica DM2000 across different fields. In at least three independent experiments, a total of at least 200 cells were counted for each treatment (approximately 20 cells were counted per field, and a total of 10 number of fields per coverslip were examined).

### Antibodies

Ki67 (1 μg/ml, catalog no.: ab15580; Abcam), Tbx20 (5 μg/ml, catalog no.: PA5-40669; Thermo Fisher Scientific), Bmp2 (1:200 dilution, catalog no.: PA5-85956; Thermo Fisher Scientific), Atf6 (1:250 dilution, catalog no.: sc-166659; Santa Cruz Biotechnology), Chop (1:500 dilution, catalog no.: 2895S; Cell Signaling Technology), pSmad1/5/9 (1:500 dilution, catalog no.: 13820S; Cell Signaling Technology), Smad1 (1:1000 dilution, catalog no.: 9743S; Cell Signaling Technology), α-SMA (1 μg/ml, catalog no.: 14-9760-82; Thermo Fisher Scientific), Bax (1:200 dilution, catalog no.: 2772S; Cell Signaling Technology), Bcl_XL_ (1:200 dilution, catalog no.: 2764S; Cell Signaling Technology), Mf20 (1:200 dilution; Developmental Studies Hybridoma Bank, University of Iowa), Grp78 (1:1000 dilution, catalog no.: 3177; Cell Signaling Technology), p-JNK (1:1000 dilution, catalog no.: 9251S; Cell Signaling Technology), JNK (1:1000 dilution, catalog no.: 9252S; Cell Signaling Technology), Col I (1:1000 dilution, catalog no.: PA5-95137; Thermo Fisher Scientific), Col III (1:1000 dilution, catalog no.: PA5-95595; Thermo Fisher Scientific), Periostin (1:1000 dilution, catalog no.: ab14041; Abcam), Runx2 (1:500 dilution, catalog no.: NBP2-67777; Novus Biologicals), Goat Anti-Rabbit IgG H&L (Alexa Fluor 488) (1:1000 dilution, catalog no.: ab150077; Abcam), Goat Antimouse IgG H&L (Texas Red) (1:1000 dilution, catalog no.: ab6787; Abcam), anti-rabbit IgG, HRP-linked Antibody (1:1000 dilution, catalog no.: 7074S; Cell Signaling Technology), antimouse IgG, HRP-linked Antibody (1:1000 dilution, catalog no.: 7076S; Cell Signaling Technology).

### ECG recording in anesthetic rats

Male Wister rats treated with Tun for different time intervals were anesthetized using ketamine (60 mg/kg BW) and xylazine (10 mg/kg BW) as previously mentioned (70). ECG of the anesthetized rats was recorded for 10 min using standard lead II (metal ECG leads). The acquired ECG signals were analyzed by BIOPAC (Biosystems) MP36. The QT duration was measured from the onset of the QRS complex to the end of T wave. The time elapsed between two successive R waves of the QRS signal gave the measure of the RR interval.

### Determination of HW/BW ratio

HW normalized to BW was measured in milligram/gram units after euthanization using carbon dioxide, followed by cervical dislocation and harvested at 8 h and 2 days.

### Immunohistological analysis

Heart tissue from the three different groups was washed in PBS and fixed in 4% paraformaldehyde and embedded as previously described (22, 71). For histological staining, 5 μm tissue sections were deparaffinized, rehydrated, and used for subsequent analysis purpose.

### Cardiomyocyte size determination

The cross-sectional area of individual cardiomyocytes was determined by staining 5 μm tissue sections with FITC-conjugated wheat germ agglutinin (catalog no.: L4895; Sigma–Aldrich) for 1 h at room temperature. The nuclei were counterstained with nuclear stain 4′,6-diamidino-2-phenylindole (catalog no.: D9542; Sigma–Aldrich). The images were taken by Leica DM2000 across multiple fields. Cell size was quantified using ImageJ (National Institutes of Health) software.

### Fibrosis detection

Collagen deposition was determined by staining 5 μm tissue sections with Masson’s trichrome reagent. The collagen deposition was quantified using ImageJ (National Institutes of Health) software.

### Real-time qRT–PCR

Total RNA from cell and adult mouse and rat hearts was isolated using TRIzol Reagent (catalog no.: 15596026; Thermo Fisher Scientific). Following quantification using Qubit4 Fluorometer (Thermo Fisher Scientific), 1 μg RNA from each sample was used for complementary DNA (cDNA) preparation using iScript cDNA synthesis kit (catalog no.: 170889; Bio-Rad). About 1 μl of the synthesized cDNA was used for qRT–PCR using iTaq Universal SYBR Green Supermix (catalog no.: 1725121; Bio-Rad) in 7500 real-time PCR system (Applied Biosystems). The amplification was carried out using following thermal conditions initial holding at 95 °C for 10 min followed by 40 cycles of 95 °C for 15 s and 60 °C for 1 min and a dissociation stage of 95 °C for 15 s, 60 °C for 1 min, and then 95 °C for 30 s. Expression of *β-actin* mRNA was used as an endogenous control. The amount of RNA was quantified using the comparative CT method (ΔΔCt). The list of the primers used is mentioned in Table S1.

### ROS estimation

For quantification of the intracellular ROS generation because of various treatment conditions, the control and treated H9c2 cells were treated with 2′,7′-dichlorofluorescin diacetate (D6883; Sigma–Aldrich), which reacts with the intracellular ROS generated to give a green fluorescent compound dichlorofluorescein. Following different treatments, the H9c2 cells were washed with ice-cold Hanks balanced salt solutionand incubated with 100 μM dichlorofluorescein diacetate for 30 min at 37 °C. Following lysis of the cells with alkaline solution, the fluorescence intensity was measured at excitation of 485 nm and emission at 520 nm (Hitachi). For positive control, H9c2 cells were treated with 1% H_2_O_2_ for 6 h.

### ChIP assay

DNA–protein complexes in cultured H9c2 cardiomyocytes treated with 20 μg/ml Tun were crosslinked for 10 min at room temperature by adding formaldehyde (Himedia) at a final concentration of 1% to the culture media. 10× Glycine (Himedia) was added to each dish to quench unreacted formaldehyde. The fixed cells were lysed in SDS Lysis buffer and sonicated ten times for 30 s with a 1 min refractory period. The cell lysate was centrifuged 10,000*g* at 4 °C for 10 min to remove insoluble material. For immunoprecipitation, 10 μg of digested crosslinked chromatin was incubated with antibody against Atf6 (5 μg; catalog no.: sc-166659, Thermo Fisher Scientific) and incubated at 4 °C overnight. Immunoprecipitation with normal rabbit IgG was used as a negative control. Following incubation with respective antibodies, 60 μl of Protein AG Plus Agarose Beads (catalog no.: BB-PAG001PB, BioBharati) was added to each immunoprecipitate and incubated for 1 h at 4 °C. After centrifugation at 3000*g* for 1 min, the beads were washed with low salt immune complex wash buffer (one wash), high salt immune complex wash buffer (one wash), LiCl immune complex wash buffer (one wash), and TE buffer (one wash). The DNA–protein complexes were eluted in elution buffer. To free the DNA, the DNA–protein complexes were reversed crosslinked using 5 M NaCl and incubated at 65 °C for 5 h. The protein was removed by digestion with proteinase K at 65 °C for 2 h. The DNA was purified using phenol chloroform method. The immunoprecipitated and input DNA were subjected to real-time PCR using SYBR Green PCR reagent with the following primers: *rtbx20* forward: 5′-GGAAGCAGTGACGTGAGAC′ and *rtbx20* reverse: 5′-GCGACCTAAACTGTGCCT-3′ to amplify rat *tbx20* promoter region. Fold enrichment relative to IgG (negative control) was calculated from three independent experiments (n = 3) using the comparative CT method (ΔΔCt) described previously (71).

In order to decipher the binding of Tbx20 to the promoter of *atf6* gene, a similar procedure was used. For immunoprecipitation, 10 μg of digested crosslinked chromatin was incubated with antibody against Tbx20 (5 μg, catalog no.: PA5-40669; Thermo Fisher Scientific). The immunoprecipitated and input DNA were subjected to real-time PCR using SYBR Green PCR reagent with the following primers: *ratf620* forward: 5′-TCCAGTCTAACGTGTGATGCA-3′ and *ratf620* reverse: 5′-AAGAGTTAGGCTTCCCACCC-3′ to amplify rat *atf6* promoter region. Fold enrichment relative to IgG (negative control) was calculated from three independent experiments (n = 3) using the comparative CT method (ΔΔCt) described previously (71).

### Statistical analysis

All the results were calculated as mean ± SD of at least three independent experiments. Statistical analysis between experimental groups was performed using Student's *t* test for two groups and one-way ANOVA for multiple groups using GraphPad Prism 9 Software (GraphPad Software, Inc). Results with *p* < 0.05 were considered significant.

## Data availability

All data are contained within the article.

## Supporting information

This article contains supporting information.

## Conflict of interest

The authors declare that they have no conflicts of interest with the contents of this article.

## Supporting information

Supplemental Table S1, Figures S1–S3 Captions
