# Supplementary material for: ER stress induces upregulation of transcription factor Tbx20 and downstream Bmp2 signaling to promote cardiomyocyte survival
Source: J Biol Chem. 2023 Feb 16;299(4):103031. doi: 10.1016/j.jbc.2023.103031 (PMC10036653; doi:10.1016/j.jbc.2023.103031)
Supplement: Supplemental Table S1, Figures S1–S3 Captions [file mmc1.docx]

**Supporting Information**

**ER stress induces upregulation of transcription factor Tbx20 and downstream Bmp2 signaling to promote cardiomyocyte survival**

Shreya Das^a^, Arunima Mondal^a^, Chandrani Dey^a^, Santanu Chakraborty^b^, Rudranil Bhowmik^c^, Sanmoy Karmakar^c^ and Arunima Sengupta^a,*^

^a^Department of Life Science and Biotechnology, Jadavpur University, Kolkata, India

^b^Department of Life Sciences, Presidency University, Kolkata, India

^c^Bioequivalence Study Centre, Department of Pharmaceutical Technology, Jadavpur University, Kolkata, India

**Materials Included: Table S1, Figure S1, Figure S2, Figure S3**

**Table S1: List of primers**

| **Primer Name** | | **Primer Sequence (5’ 3’)** | | **Accession number** |
| --- | --- | --- | --- | --- |
| ***Rattus norvegicus*** | | | | |
| *atf6* | Forward | GTACTGAGGAGACAGCAGCG | | NM_001107196.1 |
|  | Reverse | GCCTCTGGTTCTCTGACACC | |  |
| *grp78* | Forward | TCAGCCCACCGTAACAAT | | NM_013083.2 |
|  | Reverse | CAAACTTCTCGGCGTCAT | |  |
| *tbx20* | Forward | AGGAGCTCTGGGACAAATTCC | | NM_001401090.1 |
|  | Reverse | GAACATCCTCCTGCCAGACTTG | |  |
| *bmp2* | Forward | AAGAAGCCATCGAGGAACTTCCAG | | NM_017178.2 |
|  | Reverse | CCTGAGACCAGCTGTGTTCATCTT | |  |
| *bnp* | Forward | AGTCCTAGCCAGTCTCCAGA | | NM_031545.1 |
|  | Reverse | GTCTCTCCTGGATCCGGAAG | |  |
| *β-mhc* | Forward | CCAGTCCCGAGGTGTACTTT | | NM_017240.2 |
|  | Reverse | TCCTCCTTCATGTTGGCCAT | |  |
| *serca2* | Forward | GCTCCATCTGCTTGTCCATG | | NM_001110823 |
|  | Reverse | CAGGCAGGGAGATTTTCAGC |  | |
| *β-actin* | Forward | TCTTCCAGCCTTCCTTCCTG | NM_031144.3 | |
|  | Reverse | CACACAGAGTACTTGCGCTC |  |  |
| ***Mus musculus*** | | | | |
| *atf6* | Forward | GAAGTGGAAAGGACCAAATCTAGAAG | | NM_001081304.1 |
|  | Reverse | CTCACTCCCAGAATTCCTACTGATG | |  |
| *grp78* | Forward | TGCAGCAGGACATCAAGTTC | | NM_022310.3 |
|  | Reverse | TACGCCTCAGCAGTCTCCTT | |  |
| *chop* | Forward | CTGCCTTTCACCTTGGAGAC | | NM_007837.4 |
|  | Reverse | CGTTTCCTGGGGATGAGATA | |  |
| *tbx20* | Forward | AAACCCCTGGAACAATTTGTGG | | NM_194263.3 |
|  | Reverse | CATCTCTTCGCTGGGGATGAT | |  |
| *bmp2* | Forward | TGCACCAAGATGAACACAGC | | NM_007553.3 |
|  | Reverse | GTGCCACGATCCAGTCATTC | |  |
| *bnp* | Forward | AAGTCCTAGCCAGTCTCCAGA | | NM_008726.6 |
|  | Reverse | GAGCTGTCTCTGGGCCATTTC | |  |
| *β-mhc* | Forward | ACGGATGCCATACAGAGGAC | | NM_080728.3 |
|  | Reverse | CCTCATAGGCGTTCTTGAGC | |  |
| *serca2* | Forward | GGGCGAGCCATCTACAACAA | | NM_001110140.3 |
|  | Reverse | TGTCACCAGATTGACCCAGAGT | |  |
| *β-actin* | Forward | CCTCTATGCCAACACAGTGC | | NM_007393.5 |
|  | Reverse | CCTGCTTGCTGATCCACATC | |  |

**Figure S1: ER stress inducers (Tun, DTT, Tg) results in upregulation in the expression of Tbx20 and Bmp2.** (A) Western blot analysis showing a gradual increase in the expression of Tbx20 and Bmp2 upon ER stress induction with tunicamycin (Tun) for 8 hours (h) and 12 h. (B) Quantitative representation by ImageJ software of the proteins using three biological replicates from A. (C) H9c2 cells were treated with different concentration (2 μg/ml, 5 μg/ml 10 μg/ml, 20 μg/ml, 50 μg/ml and 100 μg/ml) of Tun for 24 h and cell viability was assessed by 3-[4,5-Dimethylthiazol-2-yl]-2,5 diphenyl tetrazolium bromide (MTT) assay. The cell viability reduced over a period of 24 h. The difference in cell viability between control, 2 μg/ml and 5 μg/ml Tun treated group was non-significant. Difference in cell viability between control and 10 μg/ml was significant. However, since difference in viability between 10 μg/ml and 20 μg/ml Tun treated groups was non-significant, hence we have used 10 μg/ml and 20 μg/ml Tun concentrations for further studies. The 50 μg/ml Tun treatment of H9c2 cells for 24 h reduced the cell viability to 45.6% which is close to IC_50_. We used 50 μg/ml Tun concentration along with 2 μg/ml, 5 μg/ml, 10 μg/ml Tun and 20 μg/ml Tun for further experiments in order to study the gene expression profile during high ER stress. (D) ER stress induction with DTT resulted in increase in the expression of Tbx20 and Bmp2 upto 3 mM DTT concentration. The expression of Tbx20 decreased significantly at 5 mM DTT concentration whereas the expression of Bmp2 decreased significantly at 10 mM DTT concentration. (E) Quantitative representation by ImageJ software of the proteins using three biological replicates from D. (F) ER stress induction with Thapsigargin (Tg) caused increase in the expression of Tbx20 and Bmp2 upto a 6 μM concentration of Tg. The expression of Tbx20 and Bmp2 decreased significantly at a Tg concentration of 10 μM. (G) Quantitative representation by ImageJ software of the proteins using three biological replicates from F. Statistical significance was calculated by one way ANOVA. Error bars represent S.D. from three independent biological replicates (n=3). ns, p: nonsignificant, *, p<0.05, **, p<0.005, ^***^, p<0.0005, ^##^, p<0.0001.

**Figure S2: Thapsigargin treatment is accompanied by increased cardiomyocyte proliferation and Tbx20 increases the expression of *atf6* during ER stress.**
(A) Immunofluorescence staining revealed increase in the expression of proliferative marker Ki67 in 3 μM Tg treated cells (a’) as compared to control (a). However, in the 10 μM Tg treated cells the expression of Ki67 is decreased (a’’). On the contrary, the expression of apoptotic marker Chop is increased (b’’) in 10 μM Tg treated cells as compared to control (b) and 3 μM Tg treated cells (b’). Scale bar represents 50μm. (B) Quantitative representation of panel A. (C) Western blot showing increase in the expression of apoptotic marker Chop in 10 μM Tg treated cells as compared to control and 3 μM Tg treated cells. (D) Quantitative representation by ImageJ software of the proteins using three biological replicates from C. (E) Bioinformatics analysis revealed the presence of canonical binding sequence for Tbx20 in the promoter of rat *atf6* gene (F) Chromatin immunoprecipitation (ChIP) assay followed by PCR analysis revealed that Tbx20 binds to *atf6* promoter and induces its activity during ER stress induced by Tun. (G) Tbx20 binds to the promoter of *atf6* with 7.3±1.2-fold enrichment over IgG controls during ER stress induced by Tun. (H) Western blot showing 73% downregulation in the endogenous level of Tbx20 upon knockdown with Tbx20 siRNA (I) Quantitative representation by ImageJ software of the proteins using three biological replicates from H. (J) Western blot showing 74.2% upregulation in the endogenous level of Bmp2 upon treatment with Recombinant Bmp2 protein. (K) Quantitative representation by ImageJ software of the proteins using three biological replicates from J. (L) Western blot showing 67% downregulation in the endogenous level of Bmp2 upon treatment with Noggin. (M) Quantitative representation by ImageJ software of the proteins using three biological replicates from L. Statistical significance was calculated by one way ANOVA. Error bars represent S.D. from three independent biological replicates (n=3). ns, p: nonsignificant, *, p<0.05, **, p<0.005, ^***^, p<0.0005, ^##^, p<0.0001.

**Figure S3: Prolonged ER stress results in alteration of cardiac function in rats with concomitant decrease in the expression of Tbx20.** (A) Prolonged ER stress induction (2 days) in rats results is significant increase in heart weight/body weight ratio as compared to ER stress induction for short duration (8 h) and control group. Scale bar represents 20μm (B) Western blot analysis showing decrease in the expression of Tbx20 upon prolonged ER stress (2 days) as compared to ER stress induction for 8 h. The expression of Bmp2 however increased significantly between 8 h and 2 days group. (C) Quantitative representation by ImageJ software of the proteins using three biological replicates from B. (D) qRT-PCR showing increase in the expression of *bnp* and *β-mhc* and decrease in the expression of *serca2* in 2 days ER stress group as compared to 8 h and control group. (E) Electrocardiograph (ECG) analysis showing altered cardiac function in 2 days Tun treated group with elevated ST segment as compared to 8 h and control group. (F) Electrocardiograph (ECG) analysis showing altered cardiac function in 2 days Tun treated group with increased QT interval as compared to control and 8 h group. (G) Electrocardiograph (ECG) analysis showing altered cardiac function in 2 days Tun treated group with decreased RR interval as compared to control and 8 h group. Statistical significance was calculated by one way ANOVA. Error bars represent S.D. from three independent biological replicates (n=3). ns, p: nonsignificant, *, p<0.05, **, p<0.005, ^***^, p<0.0005, ^##^, p<0.0001.
