# Supplementary figures and images for: ER stress induces upregulation of transcription factor Tbx20 and downstream Bmp2 signaling to promote cardiomyocyte survival
Source: J Biol Chem. 2023 Feb 16;299(4):103031. doi: 10.1016/j.jbc.2023.103031 (PMC10036653; doi:10.1016/j.jbc.2023.103031)

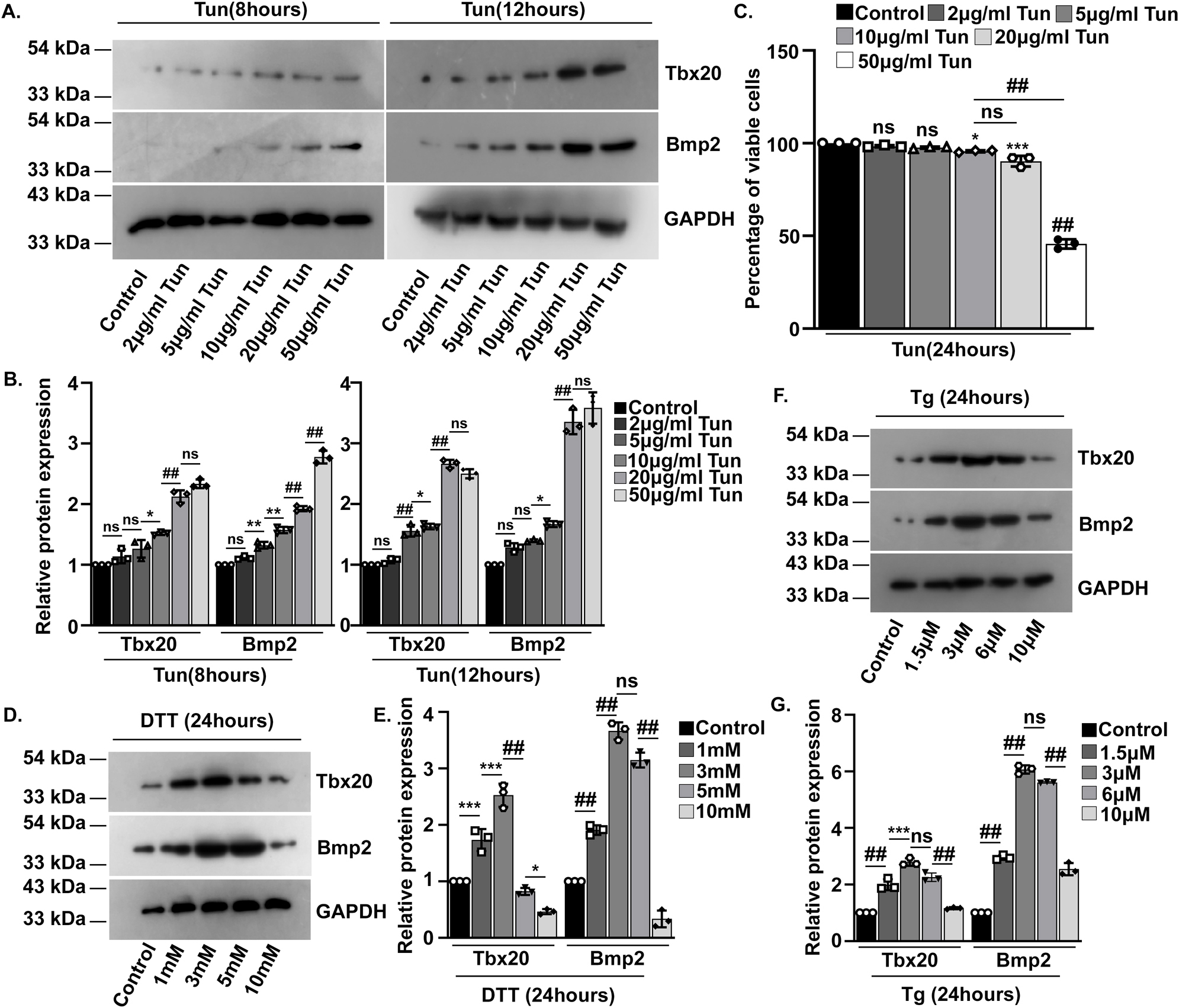

Supplement: Supplemental Figure S1 [file figs1.jpg]

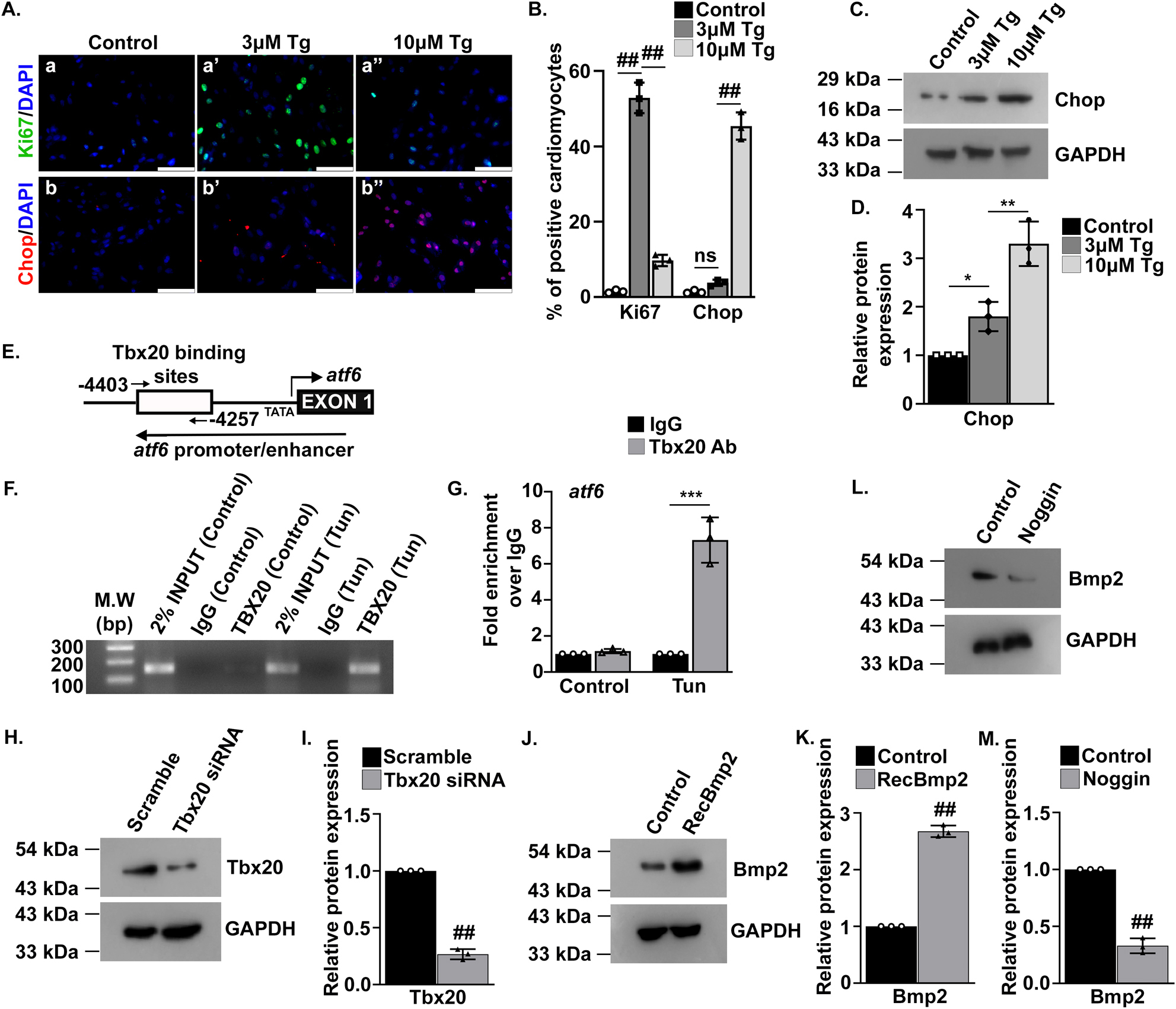

Supplement: Supplemental Figure S2 [file figs2.jpg]

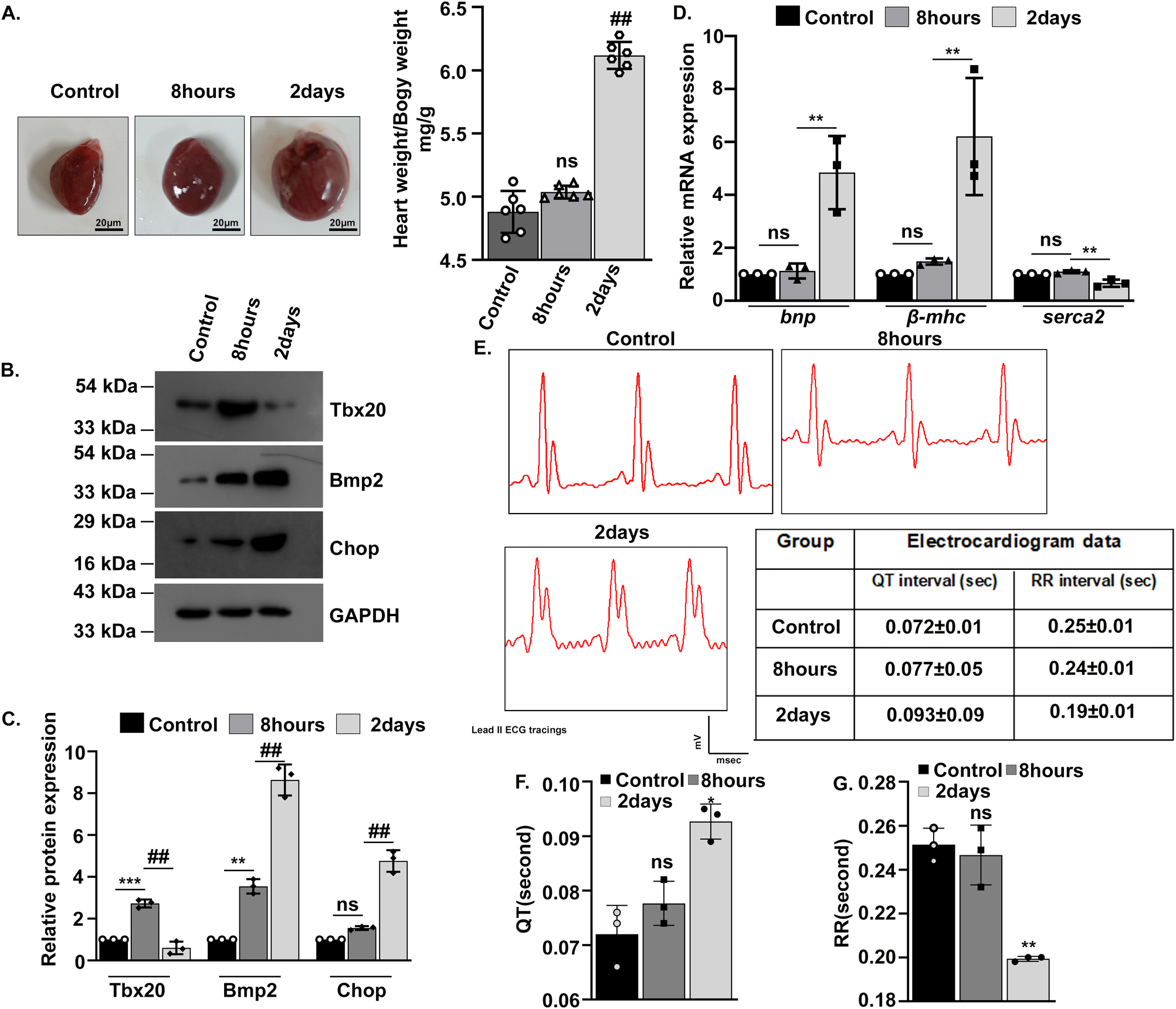

Supplement: Supplemental Figure S3 [file figs3.jpg]
